# Comparison of contact tracing methods: A modelling study

**DOI:** 10.1016/j.idm.2025.05.007

**Published:** 2025-05-16

**Authors:** Joanna X.R. Tan, Lalitha Kurupatham, Zubaidah Said, Jeremy Chan, Kelvin Bryan Tan, Marc Ho, Vernon Lee, Alex R. Cook

**Affiliations:** a Saw Swee Hock School of Public Health, National University of Singapore and National University Health System, Singapore; b Ministry of Health, Singapore; c National Centre for Infectious Diseases, Singapore

**Keywords:** Contact tracing, Forward tracing, Bidirectional tracing, Cluster, Extended tracing, COVID-19

## Abstract

**Introduction:**

Contact tracing has been a key tool to contain the spread of diseases and was widely used by countries during the COVID-19 pandemic. However, evaluating the effectiveness of contact tracing has been challenging. Approaches to contact tracing were diverse and country-dependent, with operations utilizing different tracing methods under varied environments. To provide guidance on contact tracing for future preparedness, we assessed the effectiveness of contact tracing methods under varied environments using Singapore's population structure and COVID-19 as the disease model.

**Methods:**

We developed a transmission network model using Singapore's contact tracing data and the characteristics of COVID-19 disease. We explored three different tracing methods that could be employed by contact tracing operations: forward tracing, extended tracing and cluster tracing. The forward tracing method covered the period starting two days before case isolation, the extended tracing method covered the period starting 16 days before case isolation, and the cluster tracing method combined forward tracing with cluster identification. Contact tracing operations traced detected cases from surveillance and issued interventions for identified contacts, and we constructed combinations of varied scenarios to replicate variability during pandemic, namely low case-ascertainment or high case-ascertainment and either testing of contacts or quarantine of contacts. We examined the impact of varied contact tracing operations on disease transmission and provider costs.

**Results:**

Model simulations showed that the effectiveness of contact tracing methods varied under the four different scenarios. Firstly, under low case-ascertainment with testing of contacts, contact tracing reduced transmission by 12 %–22 %, with provider costs ranging between US$2943.56 to US$5226.82 per infection prevented. The most effective tracing method to control infection was cluster tracing, followed by extended tracing and forward tracing. Secondly, under low case-ascertainment with quarantine of contacts, transmission was reduced by 46 %–62 %, with provider costs below US$4000 per infection prevented. The cluster method reduced transmission by 62 %, enough to bring the reproduction number to close to unity and was the least costly. Extended tracing reduced transmission by 50 % but costed the most, while forward tracing reduced transmission by 46 %. Thirdly, under high case-ascertainment with testing of contacts, the average transmission was reduced by 20 %–26 %, with provider costs to prevent an infection ranging between US$1872.72 to US$3165.09. There was less variability between tracing methods, with cluster tracing reducing transmission the most, followed by extended tracing and forward tracing. Lastly, under high case-ascertainment and quarantine of contacts, contact tracing was the most effective, with provider costs below US$800 per infection prevented. All tracing methods were equally effective in disease containment, bringing the reproduction number below unity and stopping disease transmission early.

**Discussion:**

We conclude that contact tracing operated most effectively when implemented with high case-ascertainment rates and quarantine of contacts; disease transmission is stopped early, and the low number of contacts enable tracing operations to be more manageable and less costly. However, the pandemic situation can be dynamic, with fluctuations in resources available for case-ascertainment and quarantine adherence, which can impact the effectiveness of contact tracing. Adapting contact tracing methods to the situation can optimize disease control. Therefore, it is recommended to develop a flexible contact tracing approach that facilitates strategy switching based on resource availability and the skills of tracing operations.

## 1 Introduction

Contact tracing (CT) has been an integral part of infectious disease surveillance and control, and combines the detection and isolation of cases, and identification of their contacts and potential testing or quarantine (Brandt, 2022). Depending on how it is implemented, disease transmission could be mitigated or even stopped. CT has been utilized to control the transmission of various infectious diseases (Fox et al., 2013), and the recommended tracing procedures vary according to the nature of the infectious disease outbreak. During the early stages of a novel outbreak, when there is still no vaccination or effective treatment, CT is an important public intervention as part of the first line of defense to stop disease spread. The most recent example was the COVID-19 pandemic, where CT was implemented globally to varying degrees of success. In some settings, CT kept the epidemic in check until vaccination could be deployed (Chow et al., 2023; Lee et al., 2021; Oshitani & Expert Members of National COVID-19 Cluster Taskforce, 2020), while in other implementation settings, it was insufficient to prevent rapid community spread (Lewis, 2020).

How CT is implemented in the context of disease epidemiology of the disease is critical for outbreak control. Generally, there are two directions that CT can be implemented, namely forward and backward. A forward directional strategy targets contacts who were infected by the case while a backward directional strategy targets the source of infection to identify further potential contacts from the source. The approaches adopted for COVID-19 varied across countries (Table 1) and depended on factors such as the local public health systems and risk assessments (Tan et al., 2025). Some worked to trace forward, such as tracing contacts from two days prior to case's onset of symptoms (ECDC, 2022; NHS, 2022). Others worked to trace in both directions by finding both infection contacts and transmission events (Dighe et al., 2020; Djuric et al., 2022; Furuse et al., 2020; PMO, 2023), or by extending period of tracing to beyond the infected individual's infectious period to capture both the upstream and downstream infections (Raymenants et al., 2022). Another unique approach was to trace generations of contacts (Nguyen et al., 2021).

**Table 1:** CT strategies for COVID-19.

Type of CT Method	Management of Contacts	Examples of Countries that utilized the strategy
Forward Tracing	Monitoring and Testing	Ireland (ECDC, 2022); Italy (ECDC, 2022); UK (NHS, 2022)
Forward Tracing	Advisory for quarantine and monitoring	Spain (ECDC, 2022)
Extended Tracing	Advisory for quarantine and monitoring	Intervention study in Belgium (Raymenants et al., 2022)
Transmission Events and Clusters	Quarantine and/or Monitoring and Testing	School Clusters in Italy (Djuric et al., 2022); South Korea (Dighe et al., 2020; Lee et al., 2021); Japan (Furuse et al., 2020); Singapore (PMO, 2023)
Generations	Direct contacts for centralized quarantine. Contacts of contacts for home quarantine.	Vietnam (Nguyen et al., 2021)

The effectiveness of CT methods in the control of COVID-19 has been assessed in several studies. Forward tracing has been shown to reduce the effective reproduction number when combined with self-isolation (Kucharski et al., 2020). Tracing backward was shown to identify more cases than tracing forward especially under conditions with overdispersion (Endo et al., 2021; Kojaku et al., 2021) and extending the period of tracing was shown to further improve disease control (Bradshaw et al., 2021; Raymenants et al., 2022). However, during the pandemic, the implementation of CT was country dependent, and CT operated under varied environments which could have affected the effectiveness of CT methods (Cook & Clapham, 2020). These varied environments included differing approaches to case finding and COVID-19 testing (Benítez et al., 2020; Haldane et al., 2021), management of identified contacts ranging from testing of contacts to legally enforced quarantine (Burke et al., 2020; ECDC, 2022; Ng et al., 2021; Salathé et al., 2020), and the availability of workforce and resources for CT (Rajan et al., 2020). There has been little evidence about the performance of CT methods under the wide range of operational environments.

To provide guidance on the future effectiveness of CT operations, we aim to simulate transmission under different CT methods and settings using Singapore's population structure and COVID-19 as our case study. Singapore conducted comprehensive CT with detailed processes to identify contacts and nationwide testing operations to detect infected individuals (Chow et al., 2023; Tan et al., 2025). Leveraging the information obtained from the comprehensive detection and tracing operations in Singapore, we first used the rich CT data from Singapore to model the potential social interactions, and then designed a model that could compare different CT methods under varied environments. By providing estimates of how disease transmission changed under the varied interventions, we aim to assist in policy decision on the utilization of CT for disease control.

## 2 Methods

### 2.1 Transmission network

#### 2.1.1 Network generation using estimates from CT data

We generated a transmission network using parameter estimates from CT data in Singapore, modeled to fit real-world interactions during the pandemic, with disease specific features. A detailed pseudocode outlining the set-up of our simulation model is available in the Supplementary.

**Fig. 1:**
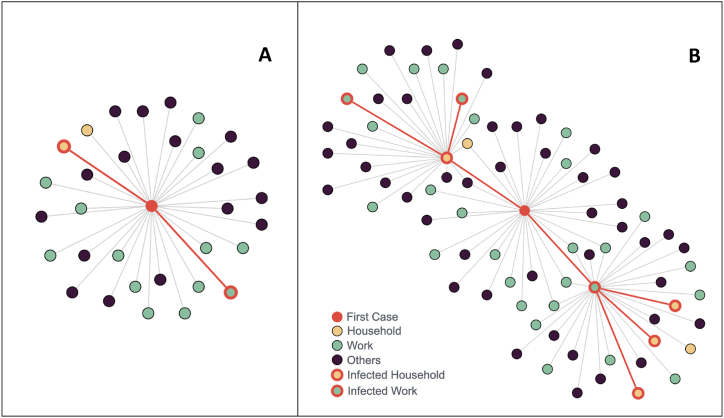
**Model of Network Structure.** (A) Contacts of one infected individual. (B) A network consisting of the first-generation infected individual from Fig. 1A, the infected second-generation individuals and their respective contacts.

We used CT data between April 2020 to June 2021, when CT was conducted for notified cases in Singapore. Based on information available in contact tracing data and given the variabilities in data entry across cases, the identified contacts of notified cases were categorized into three location categories, namely home, work or school and others. We assumed that there was no change in the number of contacts between April 2020 to June 2021. We obtained the average number of contacts in household ($\lambda_{H}$), work and school ($\lambda_{ws}$) or others ($\lambda_{o}$): $\lambda_{H}=3.4,{\;\lambda }_{ws}=8.6\;and\;\lambda_{o}=20.8$. We assumed that this was the average number of unique contacts of an individual captured by contact tracers during the infectious period (i.e. an assumption of 10 days was used in the model). Given that the contact rate between cases and contacts was not available, we assumed that household members had daily contact, work or school contacts had contact on weekday and other contacts met only one time during the infectious period. We generated a network of contacts for an individual by sampling from an assumed Poisson distribution parameterized using the estimates (Fig. 1): $C_{H}\;∼\;Poisson\;(\lambda_{H}$), $C_{ws}\;∼\;Poisson\;(\lambda_{ws}$), $C_{o}\;∼\;Poisson\;(\lambda_{o}$).

To model the relationship between cases, we included the feature of overlapping contacts in the transmission network. Singapore's contact tracing operation identified cases that are linked, and we used the linked cases to estimate the proportion of overlap. We calculated these proportions for each pair of linked cases: If Case B is a contact of Case A, let $H_{B},W_{B},O_{B}$ be the household, work or school and other contacts of Case B, and $H_{A},W_{A},O_{A}$ be the household, work or school and other contacts of Case A. The overlap proportions were calculated as follows: $P_{HH}=\frac{H_{A}\cap H_{B}}{H_{B}}$, $P_{HW}=\frac{H_{A}\cap W_{B}}{W_{B}}$, $P_{HO}=\frac{H_{A}\cap O_{B}}{O_{B}}$, $P_{WH}=\frac{W_{A}\cap H_{B}}{H_{B}}$, $P_{WW}=\frac{W_{A}\cap W_{B}}{W_{B}}$, $P_{WO}=\frac{W_{A}\cap O_{B}}{O_{B}}$, $P_{OH}=\frac{O_{A}\cap H_{B}}{H_{B}}$, $P_{OW}=\frac{O_{A}\cap W_{B}}{W_{B}}$, $P_{OO}=\frac{O_{A}\cap O_{B}}{O_{B}}$. The mean proportions of overlap between cases linked by each specific category of household, work or school and others were then calculated. These estimates were adjusted to account for potential underestimation (Supplementary Table 1) and were then included in the model.

#### 2.1.2 Disease characteristics

COVID-19 was the modeled disease in this study, and because it evolved throughout the pandemic, we focused on its early disease characteristics in 2020 (Table 2). Given that the wide heterogeneity of COVID-19 basic reproduction number estimates, we used the pooled $R_{0}$ estimate of 2.66 for our model (Dhungel et al., 2022). Other disease parameters were obtained from existing literature including incubation period from a lognormal distribution (McAloon et al., 2020), an infectious period of 10 days (Puhach et al., 2023), and asymptomatic or sub-clinical proportion at 44.1 % (Wang et al., 2023). Infected individuals without symptoms may be less infectious, and there was a wide range of proportion regarding the relative infectiousness (Buitrago-Garcia et al., 2022). We assumed a 50 % reduction in infectiousness of asymptomatic individuals, similar to other modelling studies (Johansson et al., 2021; Kucharski et al., 2020).

**Table 2:** Summary of parameters used in the model.

Parameter	Value	Source
**Network Generation**		
Average number of unique contacts	Supplementary 1*;*Supplementary Table 1	Estimated using Singapore's contact tracing data between April 2020 to June 2021.
Proportion of contacts overlap between cases	Supplementary 2*;*Supplementary Table 2	Estimated using Singapore's contact tracing data between April 2020 to June 2021.
**Disease Characteristics**		
Basic reproduction number	2.66	Dhungel et al. (2022)
Incubation Period	Lognormal distribution with mu (*1.63*) and sigma (*0.5*)	McAloon et al. (2020)
Infectious Period	10	Derived from on maximum isolation of live COVID-19 virus, which was 10 days (Puhach et al., 2023)
Degree of Infectiousness	Supplementary 3*;*Supplementary Table 3	Calculated with reference to viral load model (Puhach et al., 2023)
Probability of Asymptomatic	44.1 %	Wang et al. (2023)
Reduction in infectiousness of asymptomatic individuals	50 %	There was a wide range of proportion regarding relative infectiousness of asymptomatic individuals (Buitrago-Garcia et al., 2022). An approximation of 50 % is used in this model.
**Case Detection**		
Days between symptoms onset and test confirmation for symptomatic cases	Negative Binomial Distribution with *r*(*1*) and *p*(*1/2.68*).	Estimated using Singapore's contact tracing data between April 2020 to June 2021.

To model infection of contacts by the infected individual, we defined the contact rate per day. We assumed that household members contacted daily, contacts from work or school met on weekdays and other contacts only met once, and that all contacts were susceptible, i.e. reflecting an early pandemic stage. Next, we assigned a probability of transmission to the infected individual per contact per day, which decreased over the infectious period (Supplementary Table 2). The average number of individuals infected by the infected individual would be 2.66 to match the $R_{0}$. We included a date feature in our network model to track transmission time by assigning a date of infection for the first infected case in the network and calculating the date of infection for each subsequently infected individual.

We generated the transmission network from the first infected individual up to seven generations of infection (Fig. 1), under the assumption that infection started from the first case and there were no infectors from outside the network (i.e. no case importation). A total of 10,000 networks were simulated.

### 2.2 Implementation of CT

#### 2.2.1 CT operations: forward, extended and cluster tracing

**Fig. 2:**
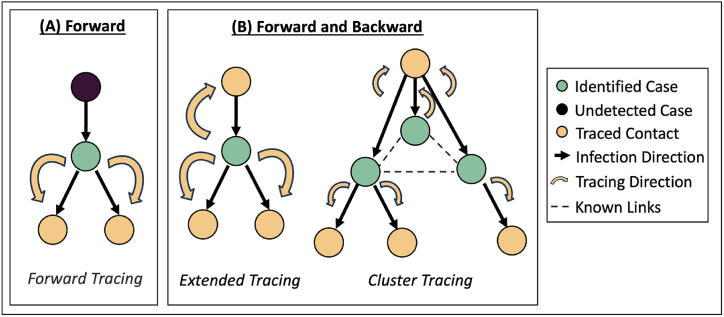
**Three types of contact tracing methods explored in model.** (A) Forward directional strategy aims to identify who the confirmed case has infected. Forward tracing method is implemented by tracing the immediate contacts of a confirmed case when the case is infectious. (B) Forward and backward directional strategy aims to identify who the confirmed case has infected and the source of infection to identify further potential contacts from the source. Extended tracing method is done by extending the period of tracing to before the case is infectious. Cluster tracing is conducted by finding individuals who are related to cluster of linked cases.

We reviewed three different CT methods: forward tracing, extended tracing and cluster tracing. These methods were modeled to mimic actual CT processes (Fig. 2). There were several assumptions built into the model. We assumed that prior to being traced, a case needs to be detected by the authorities via confirmation of COVID-19 infection. CT would begin from the first detected case rather than from the first infected case within the network. We assumed that all detected cases were strictly isolated from other contacts, similar to the practice in Singapore (Dickens et al., 2020; Nam et al., 2021). We assumed no delay in the CT process, with case identification, CT and contact notification occurring within the same day. This assumption aligned with Singapore's CT experience. Established reporting channels enabled cases to be notified immediately from healthcare institutions and laboratories to contact tracers, and efficient manpower allocation combined with the support of digital tools facilitated an immediate CT response (Tan et al., 2025; World Health Organization, 2018). Estimation for manpower hours was derived from first author's experience leading a CT team.

We defined forward CT as tracing from two days before case isolation to the day of case isolation. This tracing method was similar to the WHO recommendation for CT of cases without symptoms (WHO, 2022). The forward CT process was as follows: We detected the first confirmed case within the network based on the earliest date of case-ascertainment and isolated for ten days upon detection. We then gathered the case's information on the past movement history to find contacts within the forward CT period. The above process was estimated to take five manpower hours if performed by a contact tracer. Identified contacts would be contacted and followed up accordingly, as described under Section 2.2.2. The CT process was carried out for the next case with the earliest case-ascertainment date, and continued until all detected cases within the network were traced.

We defined extended CT as tracing from 16 days before case isolation to the day of case isolation. This approach aimed to trace the source of infection by covering the infectious period and most of the variability in the incubation period. The process was the same as forward CT, with the only difference being the extension of CT period to identify more contacts.

We defined cluster CT as a combination of forward tracing and cluster identification, designed to capture multiple transmission events. A sporadic case would indicate an unlinked or imported occurrence, while two cases with known interactions would suggest a single transmission event. The presence of three or more cases with shared epidemiological links would indicate that more than one transmission episodes have occurred within the same location. Therefore, in this model, three or more cases were used for cluster declaration. This tracing method aimed to find cluster of three or more cases with the same epidemiological link, defined as transmission occurring within the same contact category of household, work and school or others in this model. We first performed forward CT for the first detected case as per the forward CT process. Each case was then reviewed for epidemiological links with other detected cases. Once there were three or more detected cases with the same linkage (e.g. same workplace), a cluster was declared and individuals with the same epidemiological link were traced and tested. We estimated that it would take approximately 48 man-hours per cluster on average, e.g. a team of six people would take 8 h to trace the identified cluster. The CT process was carried out until all detected cases within the network were traced.

#### 2.2.2 Varied scenarios: case-ascertainment and contacts follow-up

Given that case-ascertainment and management of contacts could influence the effectiveness of CT operations, we built four combinations of scenarios, namely 1) low case-ascertainment and testing of contacts, 2) low case-ascertainment and quarantine of contacts, 3) high case-ascertainment and testing of contacts and 4) high case-ascertainment and quarantine of contacts. The three CT methods, namely forward tracing, extended tracing and cluster tracing, were evaluated within each scenario.

We first modeled variations in case-ascertainment rates to mimic the pandemic situation. Case-ascertainment rates varied during the pandemic and could be affected by factors such as sensitivity and specificity of surveillance case definitions, mandatory testing policies, and clinical assessment and guidance. We devised two baseline case-ascertainment scenarios, namely a low case-ascertainment scenario with 25 % probability of an infected individual being detected, and a high case-ascertainment scenario with 75 % probability of an infected individual being detected. We also assumed a delay from the first day of being infectious to the day of case confirmation to represent possible delays in getting detected after becoming infectious. In our model, asymptomatic individuals would have a delay of seven days, approximated by possible routes of testing and detection for asymptomatic individuals, such as weekly routine testing, and symptomatic individuals would have a delay based on an estimated marginal distribution of health seeking behavior using CT data (Table 2).

Next, we modeled the actions taken for contacts after being identified by tracers. Two approaches for the follow-up of contacts were defined. Under “Testing of Contacts” approach, identified contacts were contacted and sent for testing directly. We estimated that a contact tracer would require 10 min per identified contact. Under the “Quarantine of Contacts” approach, identified contacts were contacted and sent for a mandatory ten-day quarantine, starting from the day of contact identification. Contacts with existing quarantine had their quarantine period extended, with the end of quarantine recalculated from the last contact identification date. We estimated that a contact tracer would require 30 min per identified contact. We assumed that while under quarantine, contacts who were infected would be detected, with the date of case confirmation being either the first day of quarantine or first day of infectious period, whichever was earlier. We accounted for the infectious phase in the model. Transmission potential was reduced if the infected contact was quarantined or ascertained as a case and isolated within the infectious period, and the adjustment was based on the date of quarantine or isolation.

#### 2.2.3 Comparison of outcomes: transmission and associated provider costs

We explored how these varied CT operations affected disease transmission by reviewing the average secondary infections per case and infections transmitted within the simulated networks. We reported the mean and 95 % interval of various relevant outcomes across all the simulations, with the 95 % interval being derived from the 2.5th and 97.5th percentiles of the Monte Carlo simulation.

We estimated the direct resources required to implement CT operations for transmissions of up to seven generations of infection. Provider cost estimates obtained in Singapore dollars (SG$) were converted to US dollars (US$) at US$1 to SG$1.3221. Manpower costs were calculated by multiplying the median hourly income rate of US$15.30 (Ministry of Manpower Singapore, 2020) by number of manpower hours required to perform tracing. The cost to place a case under isolation or a contact under quarantine was approximated at US$1096.74, based on Singapore's rates for dedicated facilities for travelers during the pandemic. The cost of testing a contact was estimated at US$151.30. Given that cluster screening may extend beyond the individuals within our simulated network, we approximated that 150 people would be tested per cluster and US$1500 to perform operational-related costs such as screening sites. Provider costs per infection prevented was obtained through dividing the total costs by the mean number of cases reduced for each intervention.

We conducted sensitivity analyses with 12 variations of model parameters to assess if the uncertainties mattered. The detailed methodology and results are provided in the Supplementary. All code was written in R (R Core Team, 2024) and C++ (ISO/IEC, 2024).

## 3 Results

**Fig. 3:**
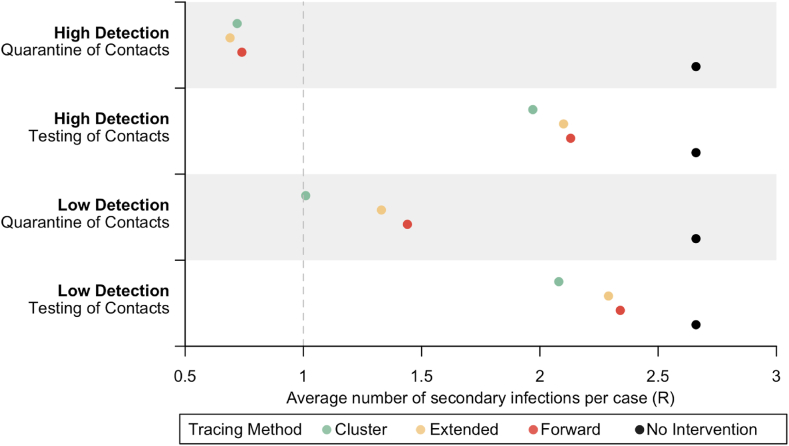
**Transmission under varied scenarios.** Each colored dot represents the reproduction number with the unique tracing method, while black dot represents no intervention. Grey line at R = 1 for reference.

**Fig. 4:**
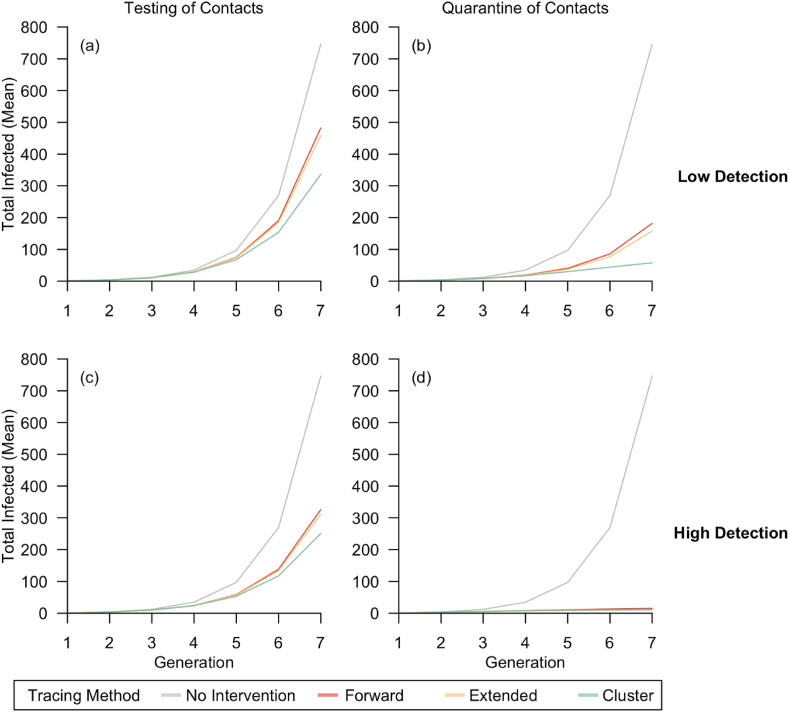
**Mean of total COVID-19 infections simulated over seven generations**. a) contact tracing with testing of contacts under low detection, b) contact tracing with quarantine of contacts under low detection, c) contact tracing with testing of contacts under high detection and d) contact tracing with quarantine of contacts under high detection.

Based on our model simulations at baseline (i.e. without any interventions), the secondary infection rate was 2.66, and 70 % of cases infected more than one individual (Table 3; Fig. 3). Each simulated network was generated with infection up to seven generations, and the baseline period of transmission between the first infected case to the last infected case in the same network was on an average of 70.5 days (Table 4; Fig. 4).

**Table 3:** Average transmission per case.

Case Detection	Contacts Follow-up	Tracing Method	% Cases with R > 1	R per case [mean, 95 % I]	% Reduction in R
	*No interventions*		70 %	2.66 [2.65–2.67]	(*Baseline*)

Low	Testing of Contacts	Forward	63 %	2.34 [2.33–2.35]	12 %
		Extended	62 %	2.29 [2.28–2.30]	14 %
		Cluster	56 %	2.08 [2.07–2.09]	22 %
Low	Quarantine of Contacts	Forward	37 %	1.44 [1.43–1.45]	46 %
		Extended	34 %	1.33 [1.32–1.34]	50 %
		Cluster	25 %	1.01 [1.00–1.02]	62 %
High		Forward	58 %	2.13 [2.12–2.14]	20 %
	Testing of Contacts	Extended	57 %	2.10 [2.09–2.11]	21 %
		Cluster	54 %	1.97 [1.96–1.98]	26 %
High	Quarantine of Contacts	Forward	18 %	0.74 [0.74–0.75]	72 %
		Extended	16 %	0.69 [0.69–0.70]	74 %
		Cluster	17 %	0.72 [0.71–0.72]	73 %

**Table 4:** Transmission for seven generations.

Case Detection	Contacts Follow-up	Contact Tracing Method	Days of Transmission [mean, 95 % I]	% Mean Reduction in Transmission Days
	*No interventions*		70.5 [70.2–70.7]	(*Baseline*)

Low	Testing of Contacts	Forward	66.4 [66.1–66.7]	6 %
		Extended	66.1 [65.8–66.4]	6 %
		Cluster	65.3 [65.0–65.6]	7 %
Low	Quarantine of Contacts	Forward	44.6 [44.1–45.2]	37 %
		Extended	41.2 [40.7–41.8]	42 %
		Cluster	34.4 [34.0–34.8]	51 %
High	Testing of Contacts	Forward	63.2 [62.9–63.6]	10 %
		Extended	62.9 [62.5–63.3]	11 %
		Cluster	62.6 [62.2–62.9]	11 %
High	Quarantine of Contacts	Forward	21.3 [21.0–21.7]	70 %
		Extended	17.5 [17.2–17.8]	75 %
		Cluster	19.3 [19.0–19.6]	73 %

For the effectiveness of CT in low case-ascertainment under “Testing of contacts” approach (Table 3; Table 4; Table 5), cluster tracing had the largest transmission reduction with average reproduction number at 2.08 per case, 65.3 days of transmission, and costed US$3041.81 per infection prevented. Extended tracing resulted in 2.29 transmission per case, 66.1 mean days of transmission and costed the most at US$5226.82 per infection prevented. Forward tracing reduced the average secondary infections to 2.34 per case with 66.4 mean days of transmission and was the cheapest method at US$2943.56 per infection prevented.

**Table 5:** Estimated resources for contact tracing operations.

Case Detection	Contacts Follow-up	Contact Tracing Method	Operational Resource	Interventions Performed			Estimated Costs per Infection Prevented (US Dollars)
			Total Manpower Hours [mean, 95 % I]	Total Contacts Identified [mean, 95 % I]	Total Screened Clusters [mean, 95 % I]	Total Issued Days for Mandatory Isolation [mean, 95 % I]	
Low	Testing of Contacts	Forward	1875 [1845–1904]	1709 [1682–1736]	–	4452 [4381–4522]	$2943.56
		Extended	2812 [2767–2856]	5959 [5864–6053]	–	5092 [5011–5173]	$5226.82
		Cluster	2757 [2717–2797]	2041 [2011–2072]	16 [16–16]	4601 [4535–4667]	$3041.81
Low	Quarantine of Contacts	Forward	587 [573–602]	468 [457–478]	–	5982 [5845–6120]	$1304.61
		Extended	1010 [985–1035]	1369 [1336–1402]	–	15100 [14734–15466]	$3195.09
		Cluster	682 [666–698]	516 [504–528]	4 [4–4]	6176 [6033–6318]	$1242.53
High	Testing of Contacts	Forward	1869 [1838–1900]	1840 [1810–1870]	–	4376 [4303–4448]	$1872.72
		Extended	2501 [2459–2543]	5703 [5609–5797]	–	4341 [4268–4414]	$3165.09
		Cluster	2122 [2090–2154]	1612 [1587–1637]	13 [12–13]	3495 [3442–3548]	$1947.59
High	Quarantine of Contacts	Forward	152 [148–157]	164 [160–169]	–	1953 [1897–2010]	$330.46
		Extended	241 [234–248]	385 [374–395]	–	4138 [4019–4256]	$701.09
		Cluster	163 [159–167]	136 [132–139]	1 [1–1]	1594 [1557–1631]	$298.57

For CT operations conducted under low case-ascertainment with “Quarantine of contacts” approach (Table 3; Table 4; Table 5), cluster tracing was the most effective method and reduced transmission by 62 %, enough to bring the reproduction number to close to unity. Only a quarter of cases infected more than one individual and the transmission period between the first and last case was reduced by half to an average of 34.4 days and costed the least at US$1242.53 to prevent one infection. Extended tracing was the second most effective method and reduced transmission by half, with mean transmission days of 41.2, and costed US$3195.09 per infection prevented. Forward tracing had an average secondary infection rate of 1.44, mean period of transmission at 44.6 days, and costed US$1304.61 per infection prevented.

CT methods performed under high case-ascertainment with testing of contacts had less variability (Table 3; Table 4; Table 5). Cluster tracing reduced transmission the most, with average reproduction number at 1.97 and costed US$1947.59 per infection prevented. Forward tracing and extended tracing reduced the average reproduction number from baseline of 2.66 to 2.10 and 2.13 respectively. The provider cost to implement extended tracing was US$3165.09 per infected prevented, double that of forward tracing at US$1872.72.

CT operations were optimal with high case-ascertainment and quarantine of contacts, where the reproduction number dropped to below unity for all methods. All tracing methods were equally effective in disease containment under this scenario, reducing transmission by at least 70 % (Table 3). Under this scenario within the simulated networks, all tracing methods stopped disease transmission early and transmission did not continue beyond 22 days on average (Table 4; Fig. 6). As infection was stopped early with low number of infected individuals, there were fewer manpower hours required for tracing operations and fewer interventions required to be performed, translating to a provider cost of US$298.57 to US$701.09 per infection prevented (Table 4).

**Fig. 5:**
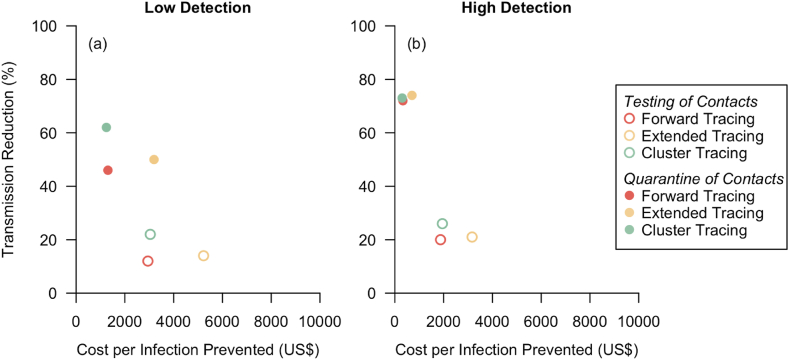
**Cost effectiveness of contact tracing methods.** a) low case detection and b) high case detection.

In general, case-ascertainment and management of contacts affected the effectiveness of CT (Fig. 3; Fig. 4; Fig. 5). A high proportion of case ascertained enhanced the effect of CT in disease control; there were 4 %–26 % more infections reduced under high case-ascertainment as compared to low case-ascertainment. The effectiveness of CT methods varied less under high case-ascertainment; there was up to 16 % variability in transmission reduction across CT methods in low case-ascertainment as compared to 6 % variability in high case-ascertainment. CT conducted with quarantine of contacts outperformed testing of contacts. By quarantining contacts, there was 34 %–53 % more transmission reduced as compared to testing of contacts.

**Fig. 6:**
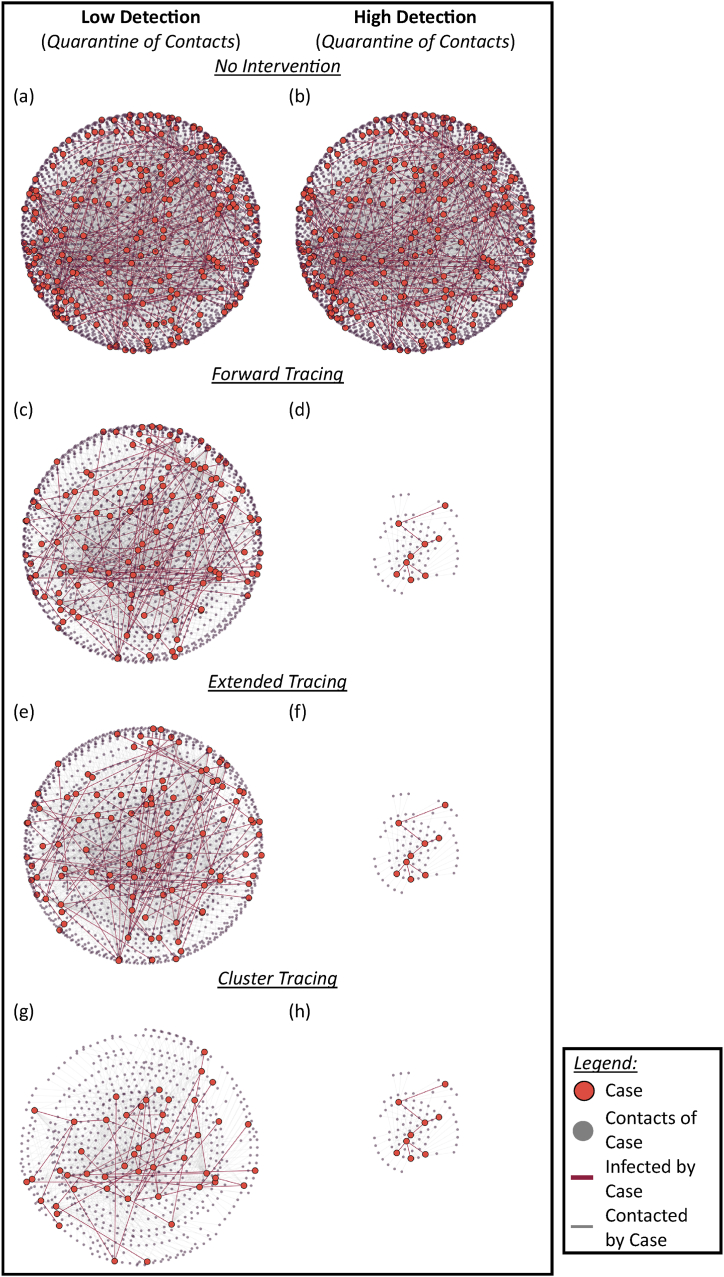
**Visualization of a simulated transmission network for seven generations, under different tracing methods with quarantine of contacts.** The full transmission network of actual contact links and infection between cases and contacts of cases as represented in (a) and (b), if there were no interventions applied. Implementing contact tracing through case isolation and contacts quarantine prevented contact between cases and contacts and reduced the number of infected cases in both low detection scenario (c), (e), (g) and high detection scenario (d), (f), (h).

We tested the sensitivity of our model to the variabilities of model parameters under high case-ascertainment, and the findings remained relatively similar across varied parameters (details in the supplementary material).

## 4 Discussion

Our findings provide insight into the variability of the effectiveness of CT operations. Our model explored tracing operations with varied CT methods, including detecting cases and issuing interventions for identified contacts. Our findings showed that higher case-ascertainment rates and quarantine of contacts increased the overall effectiveness of CT operations while reducing provider costs. Quarantine of contacts was especially effective in reducing transmission and the growth of infection. We conclude that CT operations were optimal when implemented with quarantine of contacts under high case-ascertainment rates. Under this ideal combination, disease transmission is stopped early as infected individuals are identified and isolated quickly. The low number of cases enable CT operations to be more manageable and less costly. Forward, extended and cluster tracing worked equally well to reduce the average infection per case to below one.

However, CT operations may have to perform under situations where the ideal scenario of high case-ascertainment and quarantine is not possible. This could occur when testing is reduced due to supply and workforce shortages (Cornish et al., 2023) or when quarantine becomes increasing difficult to implement due to factors such as low adherence (Carlsen et al., 2020) or enforcement difficulties (Parker et al., 2020). In such situations, it would be beneficial to optimize tracing operations by switching CT methods while considering resource constrains. This is especially so under low case-ascertainment rates where more variability was observed between different tracing methods. Our findings showed that the extended tracing and cluster tracing performed better than forward tracing in reducing transmission. These findings are consistent with other modelling studies which found that bidirectional tracing strategies enabled greater detection of infected individuals (Bradshaw et al., 2021; Endo et al., 2021; Kojaku et al., 2021; Raymenants et al., 2022). Our simulations found that cluster tracing was more efficient than extended tracing in reducing transmission and was less costly. A possible explanation may be that the extension of tracing period could find a few cases including the infector, but tracing clusters could potentially detect (and isolate) more infected individuals who may not have direct contact with identified case. It is important to note, however, that bidirectional tracing methods are more complex and would require resources beyond the provider costs factored into our model, such as prior training and manpower with specialized skillsets, which may not be available within a short timeframe.

Our findings may help us to understand the success and challenges of CT operations during the COVID-19 pandemic. Japan and the Republic of Korea had success in containing infections during the early pandemic using an approach that emphasized cluster investigations (Furuse et al., 2020; Lee et al., 2021). Our findings suggest that tracing clusters with quarantine was able to reduce the average number of secondary infections per case to close to unity, even under the low case-ascertainment scenario, thus enabling the containment of infection. Some countries had implemented both CT and quarantine, and experienced differing periods of successful containment and sudden increase in infections despite no change in virus properties (Rajatanavin et al., 2021; Tan et al., 2021). Our simulations suggest that one reason may be due to undetected cases and underlying transmission. We recommend shifting the focus to bidirectional tracing methods, such as extended tracing or cluster tracing, and increasing case-ascertainment rates to increase the effectiveness of CT. Other countries may have implemented CT programs with high testing rates but found that COVID-19 continued to spread, and tracing operations were costly (Wise, 2021). In such situations, it may be useful to mandate the quarantine of contacts to prevent further transmission, should the legal setting permit it. The effectiveness of CT in containing infection was debated during the COVID-19 pandemic (Juneau et al., 2023). Our findings showed that all tracing methods led to transmission reduction by at least 12 %, but at this level, CT would need to be implemented in conjunction with other public health and social measures to prevent outbreaks. Our simulation was modeled based on a $R_{0}$ of 2.66. COVID-19 had evolved over time with differing variants (Forchette et al., 2021). If the basic reproduction number was higher, we would expect the effectiveness of CT to differ more between the scenarios, with greater difficulty of disease control under the situation of low case-ascertainment rates and no quarantine of contacts.

These results have important implications for the development of CT policies for pandemic preparedness. The effectiveness of CT will be subject to the epidemiology of the outbreak, which could change rapidly. As such, we hope that our study could provide options for the development of flexible CT policies and facilitate strategy switching based on resource availability and skills of the tracing operations. Resources and related legislation to support case-ascertainment, isolation and quarantine could be examined. Given the difficulties for effective CT, especially cluster CT where tracers need to have overview of movement history of all cases to identify of potential clusters, it is important to have comprehensive training to familiarize officers with the different tracing techniques and to develop technical and digital tools to assist in the different forms of tracing. Effective CT is also dependent on strong cooperation with the public and other health services (Brandt, 2022; Rajan et al., 2020), and so research on means to build trust and collaboration in the local context would be fruitful.

We used Singapore's rich CT data to create a transmission network of cases and contacts, and we then modeled continual COVID-19 transmission across generations to understand how well interventions work at an individual level. This approach enabled the simulation of actual CT processes to review different strategies at a smaller scale. Our model had several assumptions. We assumed that all individuals were susceptible, to mimic COVID-19 transmission during early pandemic. We assumed that all detected cases were strictly isolated, and CT interventions operated without delay. There were several limitations. Homogeneous daily interactions were assumed as there was no data on the exact day-to-day or duration of interactions between cases and contacts. Contacts of cases were categorized into either household, work and school or others due to data limitations, which may limit the representation of an individual's complex social network. Given the high computational cost for simulation, transmission networks were generated for only up to seven generations. In our simulations, CT was initiated two days prior to the date of isolation. However, some countries might choose to commence forward CT two days before the onset of symptoms rather than from the date of isolation. This approach could lead to longer tracing periods as compared to our forward CT method, as some cases would only be detected one day or more after symptom onset. In these instances, forward CT would perform even better than our simulations suggest. In these instances, forward CT would perform even better than our simulations suggest. Our simulations modeled the ideal CT operations without delays. However, operational delays and challenges could occur in real-life implementation, resulting in CT being less effective than our estimated results. In our model, contacts identified by CT and sent for testing continued with their normal social interactions. However, in real-life situations, notified contacts could alter their behavior out of social responsibility, such as by self-imposing restrictions on their social interactions. In such instances, CT combined with testing of contacts would be more effective than our model estimates. The strength of our analysis was the stochastic nature which imparts variability to the number of contacts and infections. However, the model did not incorporate overdispersion, which is known to be important for COVID-19 (Endo et al., 2020). This is a limitation of the model. Analyses for the breakeven point at different reproduction numbers would be useful to understand the choice of tracing method at start of pandemic and could be considered for future research. We believe that our findings should be applicable to countries, or municipalities, with similar population structures and social interactions, but future work on other country demographics, on other combinations of CT methods, and on other pathogen typologies, would be valuable.

## CRediT authorship contribution statement

**Joanna X.R. Tan:** Writing – review & editing, Writing – original draft, Visualization, Methodology, Investigation, Formal analysis, Conceptualization. **Lalitha Kurupatham:** Writing – review & editing, Investigation. **Zubaidah Said:** Writing – review & editing, Investigation. **Jeremy Chan:** Writing – review & editing. **Kelvin Bryan Tan:** Writing – review & editing. **Marc Ho:** Writing – review & editing. **Vernon Lee:** Writing – review & editing. **Alex R. Cook:** Writing – review & editing, Supervision, Methodology, Conceptualization.

## Ethics approval

Ethics approval for this project was reviewed and obtained from the National University of Singapore (NUS-IRB-2023-775). Permission to access the data for this study was approved by the Ministry of Health, Singapore, with data collected under the Infectious Diseases Act and the COVID-19 (Temporary Measures) Act. The data used was anonymized prior to analysis. In using the digital contact tracing application, users accept the privacy safeguards and terms of use which can be found at: https://www.tracetogether.gov.sg/common/privacystatement.

## Funding

This work was supported by the Singapore Ministry of Health's 10.13039/501100001349National Medical Research Council under its National Epidemic Preparedness and Response R&D Funding Initiative (MOH-001041) Programme for Research in Epidemic Preparedness And REsponse (PREPARE).

## Declaration of competing interest

The authors declare that they have no known competing financial interests or personal relationships that could have appeared to influence the work reported in this paper.
