# Supplementary material for: Comparison of contact tracing methods: A modelling study
Source: Infect Dis Model. 2025 May 16;10(3):1020–32. doi: 10.1016/j.idm.2025.05.007 (PMC12688059; doi:10.1016/j.idm.2025.05.007)
Supplement: Multimedia component 1 [file mmc1.docx]

# Supplementary Materials

## 1. Pseudocode of Simulation Model

1. **Generate the first index case with disease characteristics:**

- Generate the first infected case with:
  - Generate a *Date of Infection*.
  - Draw the *Incubation Period* from Lognormal distribution ($\mu=1.63, \sigma=0.5).$Set *Infectious Period* = 10.
  - Determine *Symptom Status*: draw symptomatic or asymptomatic with probability 0.56 and 0.44.
  - If symptomatic: Draw *Day of Medical Visit* from Negative Binomial distribution with($r=1, p=1/2.68)$. Else: Set day of medical visit = 7.
  - Compute the *Date of Case-ascertainment* = *Date of Infection* + *Incubation Period* + *Day of Medical Visit*.
  - Select whether case will be detected or not detected, based on case-ascertainment scenario. (Case will have *Day of Medical Visit* and *Date of Case-ascertainment* assigned, but will not be detected or ascertained if *Detected* = 0)
    - If low case-ascertainment scenario: assign *Detected* = 1 or *Detected* = 0 with probability 0.25 and 0.75.
    - If high case-ascertainment scenario: draw *Detected* = 1 or *Detected* = 0 with probability 0.75 and 0.25.
  - Assign: *Infected Status* = 1, *Case Traced* = 0.

1. **Generate the contacts of the first index case**

- Generate the household, work or school and other contacts of a case from Poisson distributions of $\lambda_{H}=3.4, \lambda_{ws}=8.6 and \lambda_{o}=20.8$ respectively. Generate the day of contact for the contacts in others category within the case’s *Infectious Period*.
- Assign a unique serial number for each new contact.

1. **Generated the infected contacts of the first index case**

- Create a contact-day matrix: Columns from day = *Date of Infection* – 14, to day = *Date of Infection* + 65.
- Mark weekdays within the matrix (for work/school contacts).
- For each contact type, fill in the matrix with 1 (contact) or 0 (no contact) based on: household daily contact (1 for every day in the matrix), work/school contacts (1 for weekdays) and other contacts (1 on assigned day of contact).
- Infectious probability (to match $\mathcal{R}_{0}$of 2.66):
  - If the index case is symptomatic, *Infectious Probability* = 0.0375.
  - If the index case is asymptomatic, *Infectious Probability* = 0.01875.
  - The *Infectious Probability* decreases over 10 days (**Supplementary Table 2**)
- Mark case’s infectious period within the matrix. For each day in the index’s case infectious period, select the contacts who are in contact (1) for the day, and draw infection outcome from a Binomial distribution (1, *Infectious Probability*)
- For each newly infected contact:
  - Assign *Date of Infection* = day on which contact became infected.
  - Generate the disease characteristics (similar to Step ***1***) for each new infected individual), including *Incubation Period*, *Infectious Period, Symptom Status*, *Day of Medical Visit*.

1. **Generate the contacts of the next generation cases**

- Generate the household, work or school and other contacts of a case from Poisson distributions of $\lambda_{H}=3.4, \lambda_{ws}=8.6 and \lambda_{o}=20.8$ respectively. Generate the day of contact for the contacts in “others” within the case’s *Infectious Period*. Assign a unique serial number for each new contact.
- Assign overlapping contacts:
  - For each new contact, determine if it is also a contact of the primary (index case) by drawing from binomial distribution with the assigned overlap probability (**Supplementary Table 1**).
  - If overlapping, assign the same serial number for that contact.
- Infect the newly generated contacts using the procedure in Step ***3***.
- Repeat until the desired number of generations is reached.
- Assign a unique *Transmission Location* serial number based on infector and contact category:
  - Select all cases, sort by serial number.
  - Initialize a temporary locator list to store location number, serial number and contact category. Set location counter to 1.
  - Loop through all cases: Get the infector serial number and contact category. If infector serial number, contact category and location number are in the locator list, assign that location number. Else, increase location counter by 1 and assign new location number. Add current case serial number, contact category and location number into temporary locator list.
  - For all contacts, assign the *Transmission Location* number according to contact’s case and contact category.
- This completes one simulated network.

Repeat the ***1*** to ***4*** to generate as many networks as required.

1. **Contact tracing process**

- Select all cases with *Infected Status* = 1, *Detected* = 1 and *Case Traced* = 0. Sort by *Date of Case-ascertainment.* Select the case with the earliest *Date of Case-ascertainment* for contact tracing.
- Extract all the contacts of the case. Create the following tags for each contact: *Contact Contacted* (1), *Contact Identified* (0), *Date of Testing*, *Contact Quarantine* (0), *Quarantine Start Date*, *Quarantine End Date*.
- Create a contact-day matrix for this case: Columns from day = *Date of Infection* – 14, to day = *Date of Infection* + 65. Mark daily contact for households, weekday contact for work/school and one-time contact for others.
- Assign *Date of Case-ascertainment = Date of Contact Identification.*
- For the contact tracing periods: Forward tracing period starts from two days before case isolation to the day of case isolation. Extended tracing starts from 16 days before case isolation to the day of case isolation.
- Select the matrix columns that fall within the tracing period. For each contact who is in contact with case during the tracing period: *Contact Identified* = 1.
- For the period from *Date of Case-ascertainment* to the *Date of Case-ascertainment* + 10 (representing case isolation upon case-ascertainment): Update the matrix so that there is no further contact with this case. For each contact, *Contact Contacted* = 0 if no more contact; otherwise, *Contact Contacted = 1*.
- If testing of contacts scenario: For each traced contact, set *Date of Testing* = *Date of Contact Identification*.
- If quarantine of contacts scenario: For each traced contact, set *Contact Quarantine* = 1, *Quarantine Start Date* = *Date of Contact Identification*, *Quarantine End Date* = *Date of Contact Identification* + 10 days.
- Mark the case as traced: *Case Traced* = 1.
- For Cluster Tracing:
  - Select all cases with *Case Traced* = 1. If there are 3 or more cases from the same *Transmission Location*, declare cluster.
  - Identify all individuals from the same *Transmission Location*. Set *Date of Cluster Testing* = *Date of Case-ascertainment* of latest case + 2 days (approximately 48 hours to trace a cluster).
- For each contact, select all occurrences of the contact within the network via the assigned serial number.
  - To check for multiple quarantines: Filter *Contact Quarantine* = 1 to identify all quarantine issued for the contact. Set the *First Quarantine Date*  = earliest *Quarantine Start Date*. Set the *Last Quarantine Date* = latest *Quarantine End Date.*
  - To update the infection status for contacts with *Infected Status* = 1 and *Case Traced* = 0: If *Contact Contacted* = 0 with main infector, set *Infected Status* = 0. Else if *Date of Infection* is between *First Quarantine Date* and *Last Quarantine Date*, set *Infected Status* = 0. Otherwise, no change to *Infected Status*.
  - For contacts who are still infected with *Infected Status* = 1: Calculate the start and end of infectious period.
    - If *Date of Testing* < *Date of Case-ascertainment* and *Date of Testing* within infectious period, set *Date of Case-ascertainment* = *Date of Testing*. Else, no change to *Date of Case-ascertainment*.
    - If *Date of Cluster Testing* < *Date of Case-ascertainment* and *Date of Testing* within infectious period, set *Date of Case-ascertainment* = *Date of Cluster Testing*. Else, no change to *Date of Case-ascertainment*.
    - If *First Quarantine Date* < start of infectious period, set *Date of Case-ascertainment* = Start of Infectious Period. Else if *First Quarantine Date* within infectious period and *First Quarantine Date* < *Date of Case-ascertainment* and, set *Date of Case-ascertainment* = *First Quarantine Date*. Else, no change to *Date of Case-ascertainment*.
    - Assign *Detected* = 1

Repeat ***5)*** until all infected with *Detected* = 1 and *Infected Status* = 1 have been traced.

## 2. Adjustments to Parameters

### Network Overlap

We noted that the estimate from data was lower than expected for the overlap between household contacts of household linked cases and the overlap of workplace contacts for workplace linked cases. The reason could be due to the way that the contacts were captured and recorded during contact tracing operations. To adjust for this possible underestimation, we increased the two overlap estimates by 0.5 for our model.

We conducted sensitivity analysis to compare the original and adjusted estimates. We used the high detection scenario with cluster tracing and contacts quarantine and ran the simulation for 1,000 times. The average infection per case for the original estimates was 0.73 [95%CI: 0.71 – 0.75] and remained close to our study findings which used the adjusted estimates.

Supplementary Table 1: Proportion of Contacts Overlap between Linked Cases

|  | **Probability of Overlap with A’s Household** | **Probability of Overlap with A’s Work or School** | **Probability of Overlap with A’s Others** |
| --- | --- | --- | --- |
| **Case B is a “household” contact of Case A.** | | | |
| B’s Household | 0.4  (*0.9 was used in model*) | - | 0.04 |
| B’s Work or School | - | - | - |
| B’s Others | 0.03 | - | 0.03 |
| **Case B is a “work or school” contact of Case A.** | | | |
| B’s Household | 0.04 | - | - |
| B’s Work or School | 0.05 | 0.1  (*0.6 was used in model*) | 0.06 |
| B’s Others | - | - | 0.03 |
| **Case B is a “other” contact of Case A.** | | | |
| B’s Household | - | - | 0.04 |
| B’s Work or School | - | - | - |
| B’s Others | 0.03 | - | 0.1 |

### Infectiousness of an infected individual

We modelled the infection of contacts by an infected individual using probability of infection per day. Each contact that the case met would have a chance of getting infected on a per day basis. The total number of individuals infected by one case would be 2.66 on average.

We noted that the COVID-19 viral load showed a decreasing trend over time and the infected individuals could decrease in infectiousness over time (Puhach et al., 2023). To model the decrease in infectiousness over the infectious period, we extracted the log viral load copies from existing literature (Puhach et al., 2023). We took the base as day 5 and divided all by 5.4 viral copies/ml to obtain a multiplication factor for varying proportion of infectiousness over 10 days.

We conducted sensitivity analysis to compare between varied and constant infectiousness over the infectious period. The average infection per case was 2.66 for both. We used the high detection scenario with cluster tracing and contacts quarantine and ran the simulation for 1,000 times. We noted that the model with constant infectiousness had similar results as varied infectiousness; the average infection per case under cluster and quarantine was also reduced to 0.72 [95%CI: 0.70 – 0.74].

Supplementary Table 2: Varying Infectiousness Across Infectious Period

| **Days** | **Log 10 (Viral copies/ml)** | **Multiplication Factor for Infectiousness** |
| --- | --- | --- |
| 1 (day of onset) | 6.3 | 1.2 |
| 2 | 6.1 | 1.1 |
| 3 | 5.9 | 1.1 |
| 4 | 5.7 | 1.1 |
| 5 | 5.4 | 1.0 |
| 6 | 5.1 | 0.9 |
| 7 | 4.8 | 0.9 |
| 8 | 4.5 | 0.8 |
| 9 | 4.2 | 0.8 |
| 10 | 3.9 | 0.7 |

## 3. Sensitivity Analyses of Applied Parameters

To determine if varied parameters would influence our findings, we conducted sensitivity analyses using 12 parameter variations (**Supplementary Table 3**). We calculated the upper and lower bound parameters by applying a 10% increase and decrease from the main model parameters for the average number of unique contacts, infectious period, probability of asymptomatic, and reduction in infectiousness of asymptomatic individuals. For the incubation period, we derived the bounds based on 95% confidence intervals of the modeled distribution as per McAloon et al. For case detection, we assumed that there is no distribution, and we set the lower bound as a constant of 1 day while upper bound as a constant of 3 days.

In each variation, only one parameter was altered while other parameters remained consistent with the main model. We simulated 1,000 networks for each variation, and implemented the three CT methods with either testing of contacts or quarantine of contacts under the high case-ascertainment scenario. A total of 12 variations were performed for 12 different parameters.

Supplementary Table 3: Varied Parameters for Sensitivity Analyses

|  | **Estimate Used in Main Model** | **Parameter for**  **Lower Bound** | **Parameter for**  **Upper Bound** |
| --- | --- | --- | --- |
| **Network Generation** |  |  |  |
| Average number of unique contacts | Household: 3.4  Work/School: 8.6  Others: 20.8 | ***Varied Parameter 1***  Household: 3.06  Work/School: 7.74  Others: 18.7 | ***Varied Parameter 2***  Household: 3.74  Work/School: 9.57  Others: 22.9 |
| **Disease Characteristics** |  |  |  |
| Incubation Period | Lognormal distribution with mu (*1.63*) and sigma (*0.5*) (McAloon et al., 2020) | ***Varied Parameter 3***  Lognormal distribution with mu (*1.51*) and sigma (0.46) (McAloon et al., 2020) | ***Varied Parameter 4***  Lognormal distribution with mu (*1.75*) and sigma (*0.55*) (McAloon et al., 2020) |
| Infectious Period | 10 (Puhach et al., 2023) | ***Varied Parameter 5***  9 | ***Varied Parameter 6***  11 |
| Probability of Asymptomatic | 44.1% (Wang et al., 2023) | ***Varied Parameter 7***  39.7% | ***Varied Parameter 8***  48.5% |
| Reduction in infectiousness of asymptomatic individuals | 50% (Buitrago-Garcia et al., 2022) | ***Varied Parameter 9***  45% | ***Varied Parameter 10***  55% |
| **Case Detection** |  |  |  |
| Days between symptoms onset and treatment for symptomatic cases | Negative Binomial Distribution with *r*(*1*) and *p*(*1/2.68*). | ***Varied Parameter 11***  1 day | ***Varied Parameter 12***  3 days |

Findings under the varied parameters remained relatively consistent with the results from the main model. For the three CT methods with testing of contacts (**Supplementary Figures 1, 2 and 3**), the average infection per case deviated from the main results by a range of -0.3 to 0.2. Less deviation was observed for the three CT methods with quarantine of contacts (**Supplementary Figures 4, 5 and 6**), and the average infection per case differed from the main results by less than 0.1.

We first examined the parameter with the largest deviation from the main model results: the average number of unique contacts. Despite being the parameter with the greatest difference, the deviations were slight. For the lower bound parameter, the average infection per case differed by -0.2 to -0.3 for CT methods with testing of contacts and by -0.04 to -0.05 for CT methods with quarantine of contacts. The upper bound parameter differed by 0.15 to 0.2 for CT methods with testing of contacts and by 0.01 to 0.03 for CT methods with quarantine of contacts. Transmission across generations indicated that the lower bound and upper bound parameters resulted in lower and higher number of total infected respectively (**Supplementary Figure 7 and 8**), while the effectiveness of CT methods remained similar to the main model.

The parameter with the second largest deviation was the days between symptoms onset and treatment. Variation in this parameter could lead to delay in CT implementation. Compared to the main model, the average infection per case deviated slightly by -0.05 for the lower bound parameter across all CT methods, and by 0.1 to 0.2 for the upper bound parameter across all CT methods. Transmission across generations had similar infection numbers in the lower bound and upper bound parameters (**Supplementary Figure 17 and 18**). The effectiveness of CT remained consistent with the main results, with cluster CT being the most effective, followed by extended and forward CT.

The infectious period had the third largest deviation. Changes in this parameter could alter transmission potential, and thus the number of infections. Compared to the main model, deviations were slight: the lower bound parameter differed by less than -0.2 for CT methods with testing, and by less than -0.02 for CT methods with quarantine. The upper bound parameter differed by less than 0.2 for CT methods with testing, and by less than 0.02 for CT methods with quarantine. Transmission across generations showed fewer infections for the lower bound parameter (**Supplementary Figure 11**) and more infections for the upper bound parameter (**Supplementary Figure 12**). The overall effect of CT over the generations remained consistent with main results.

For the other parameters, the simulated results were close to the main model results (**Supplementary Figure 9, 10, 13, 14, 15 and 16**).


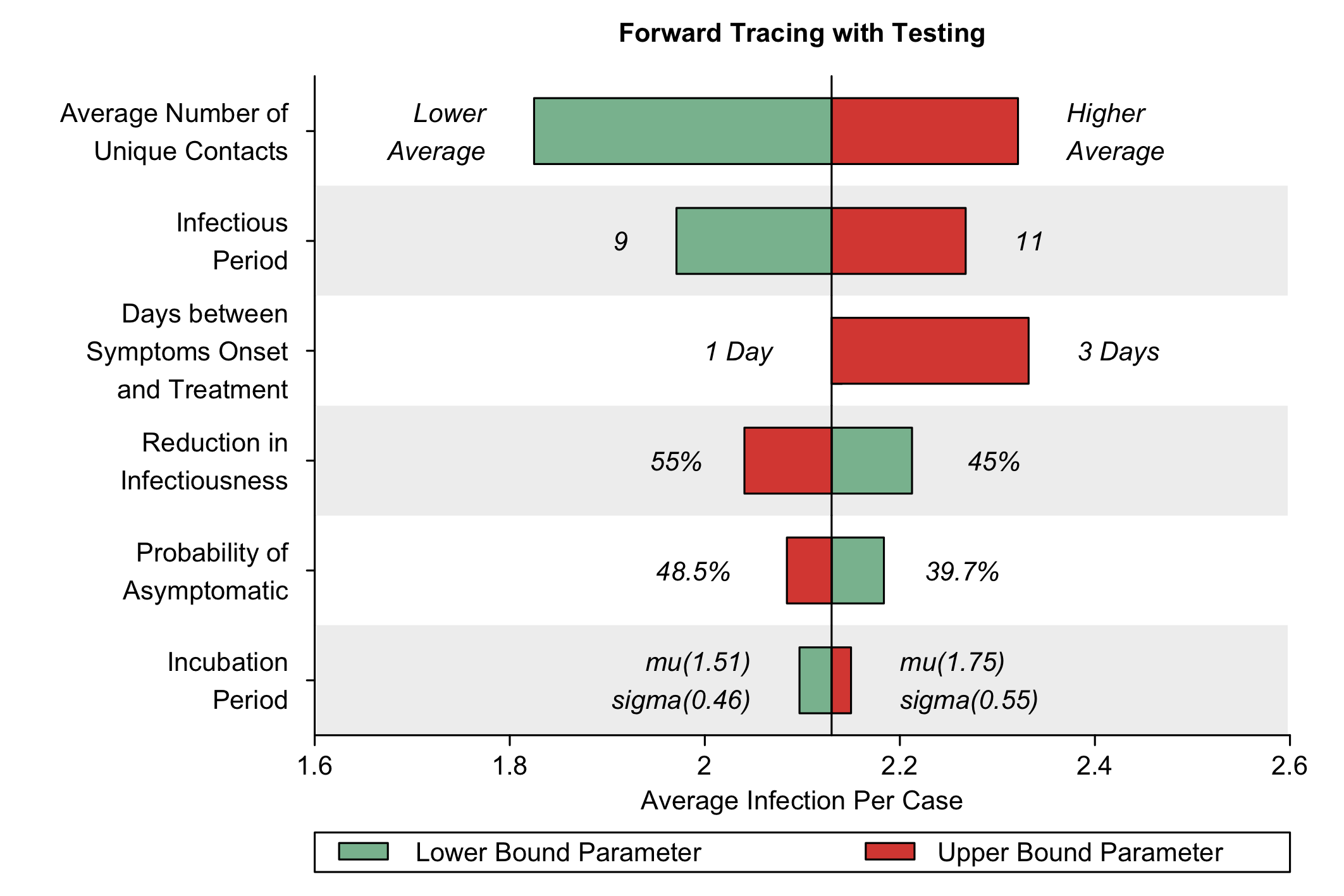


Supplementary Figure 1: Sensitivity Analysis for Forward Tracing with Testing of Contacts under 12 Parameter Variations. The black vertical line indicates the average infection per case obtained using main model parameters, while the bars represent deviations from the main results.


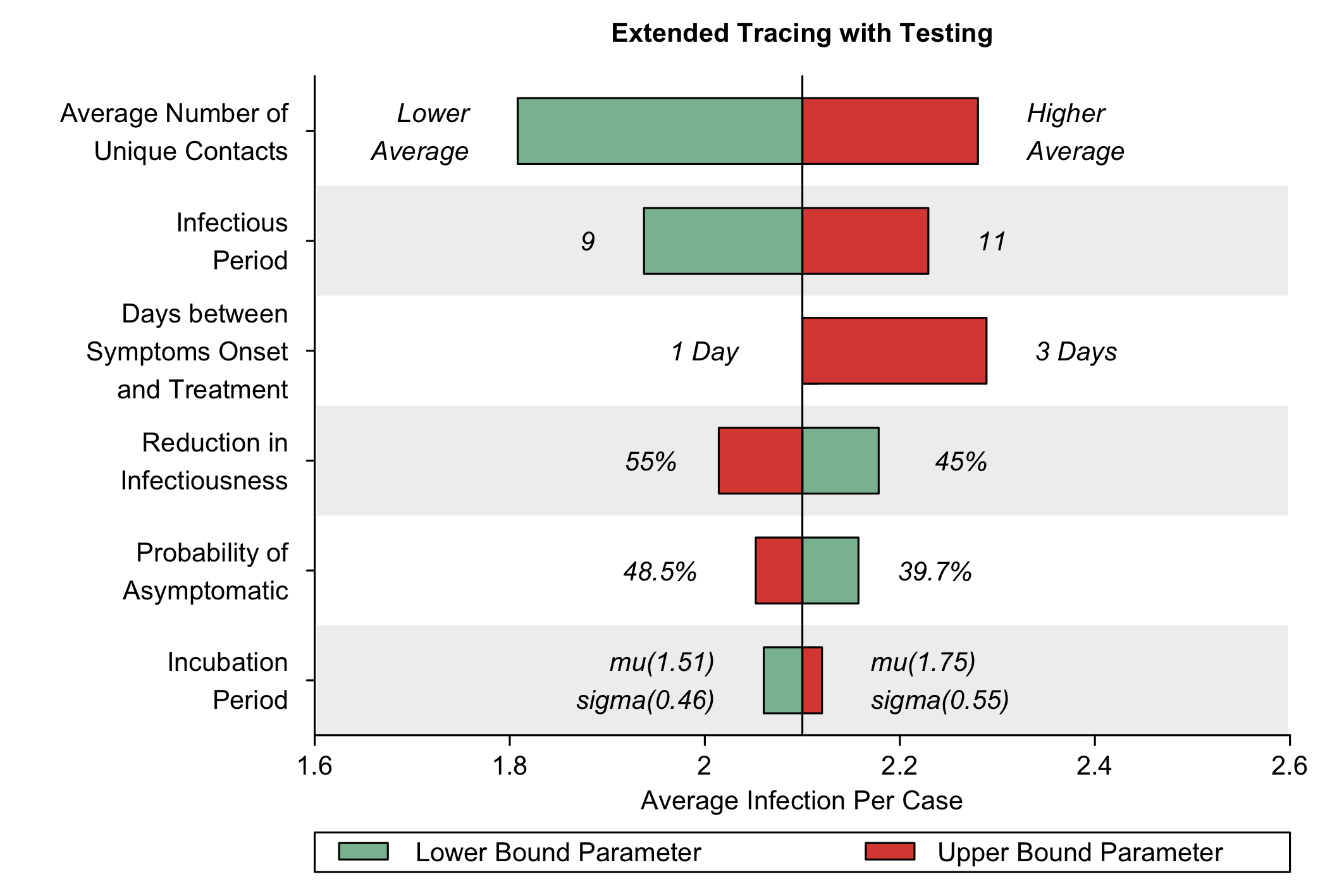


Supplementary Figure 2: Sensitivity Analysis for Extended Tracing with Testing of Contacts under 12 Parameter Variations. The black vertical line indicates the average infection per case obtained using main model parameters, while the bars represent deviations from the main results.


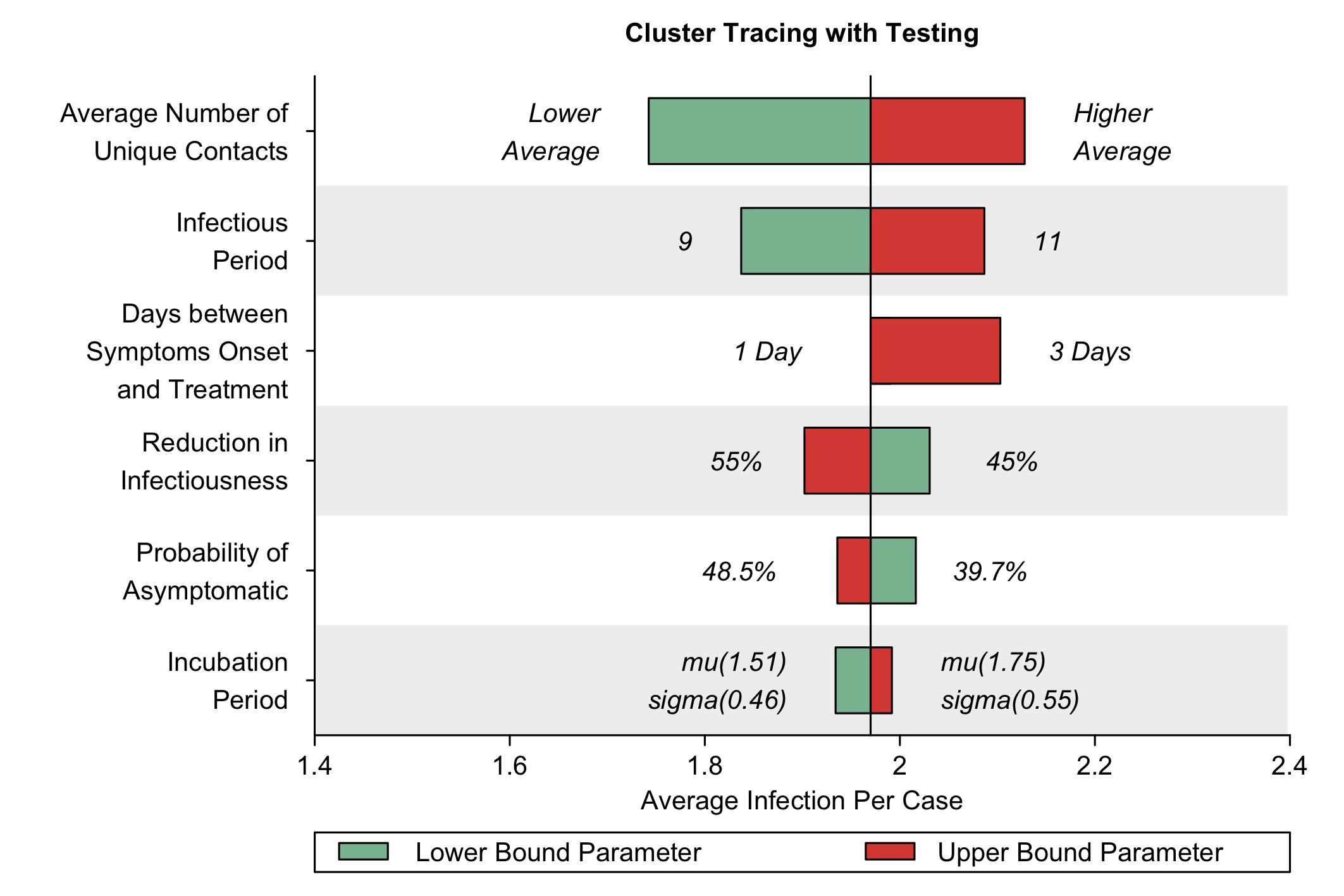


Supplementary Figure 3: Sensitivity Analysis for Cluster Tracing with Testing of Contacts under 12 Parameter Variations. The black vertical line indicates the average infection per case obtained using main model parameters, while the bars represent deviations from the main results.


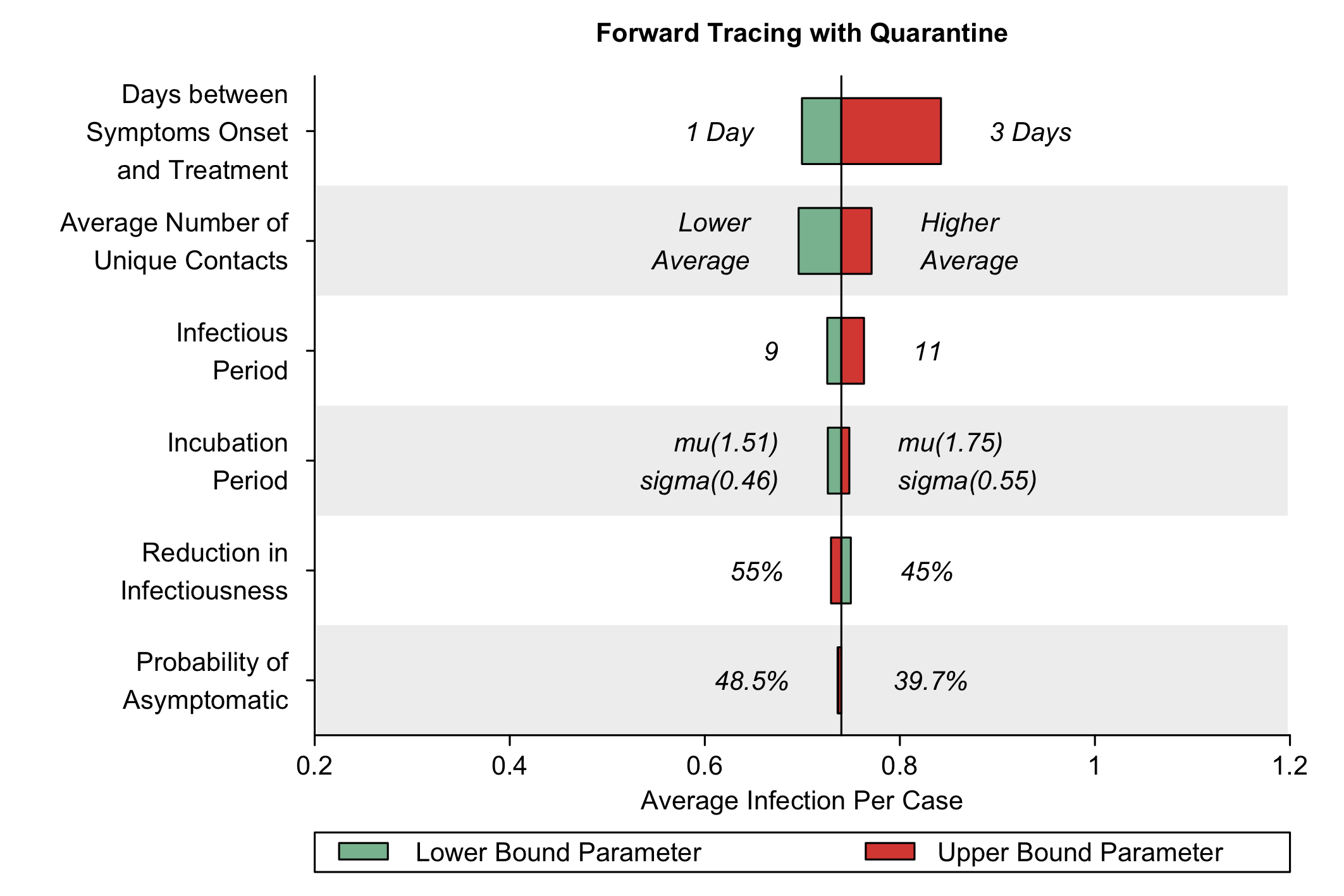


Supplementary Figure 4: Sensitivity Analysis for Forward Tracing and Quarantine of Contacts under 12 Parameter Variations. The black vertical line indicates the average infection per case obtained using main model parameters, while the bars represent deviations from the main results.


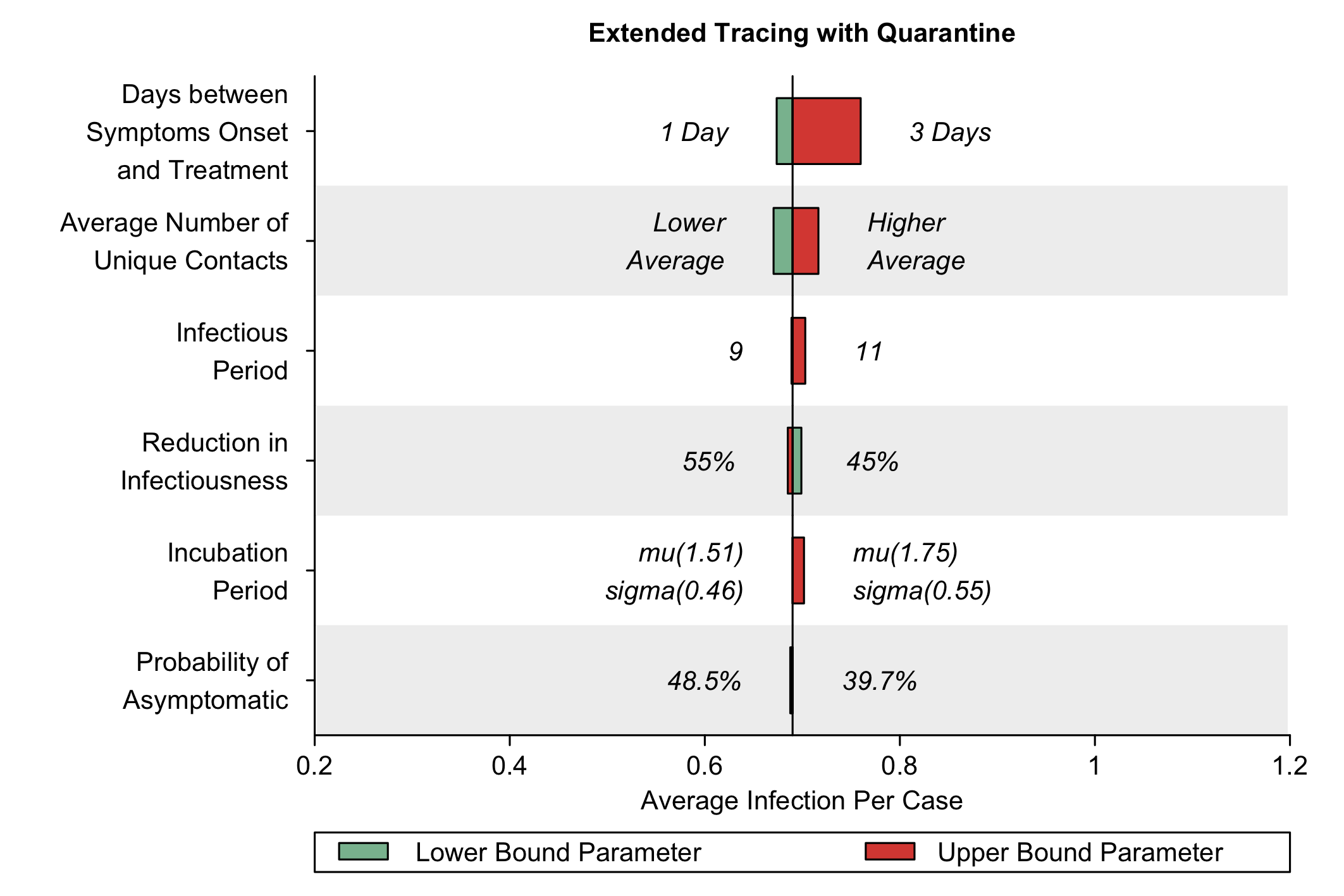


Supplementary Figure 5: Sensitivity Analysis for Extended Tracing and Quarantine of Contacts under 12 Parameter Variations. The black vertical line indicates the average infection per case obtained using main model parameters, while the bars represent deviations from the main results.


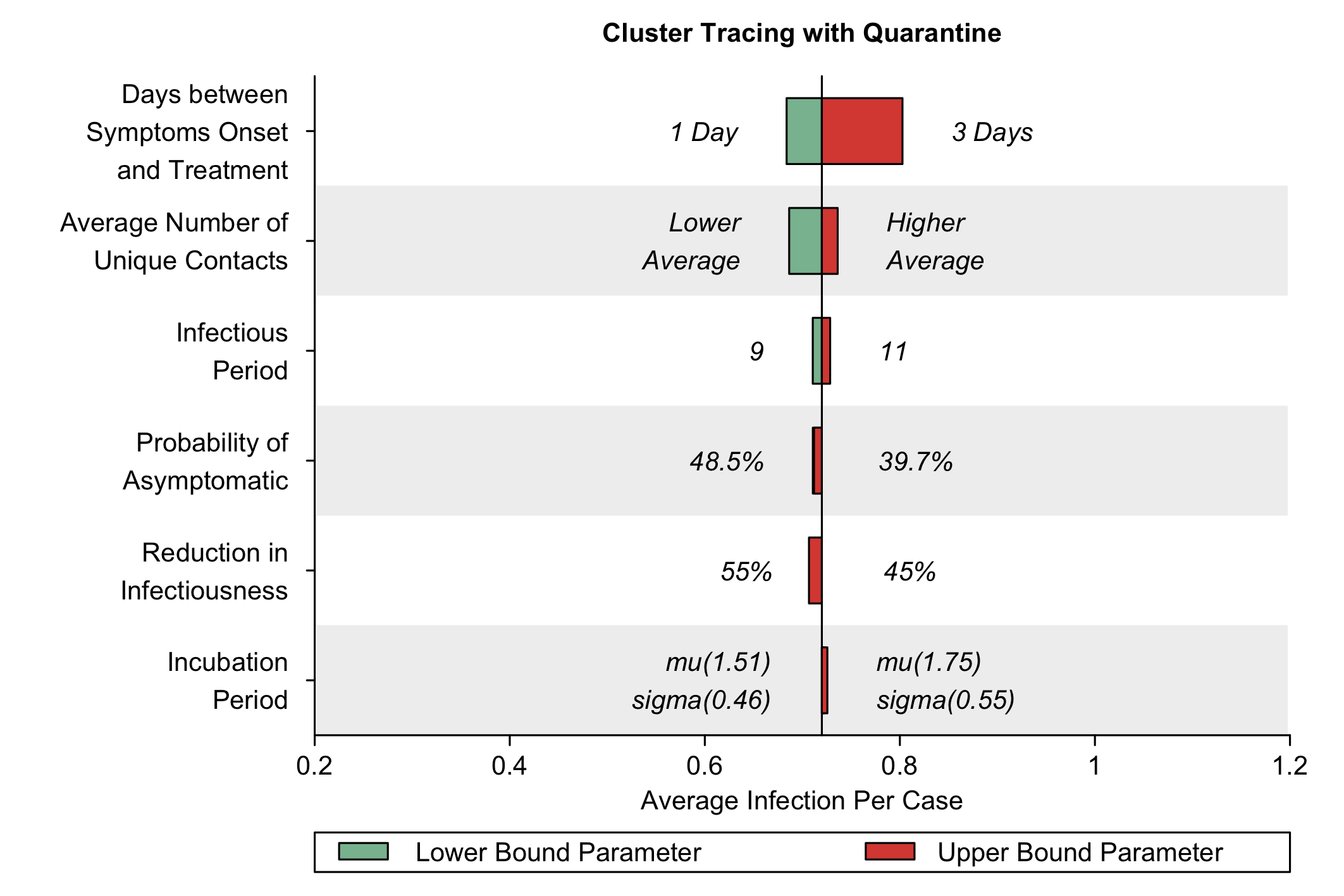


Supplementary Figure 6: Sensitivity Analysis for Cluster Tracing and Quarantine of Contacts under 12 Parameter Variations. The black vertical line indicates the average infection per case obtained using main model parameters, while the bars represent deviations from the main results.


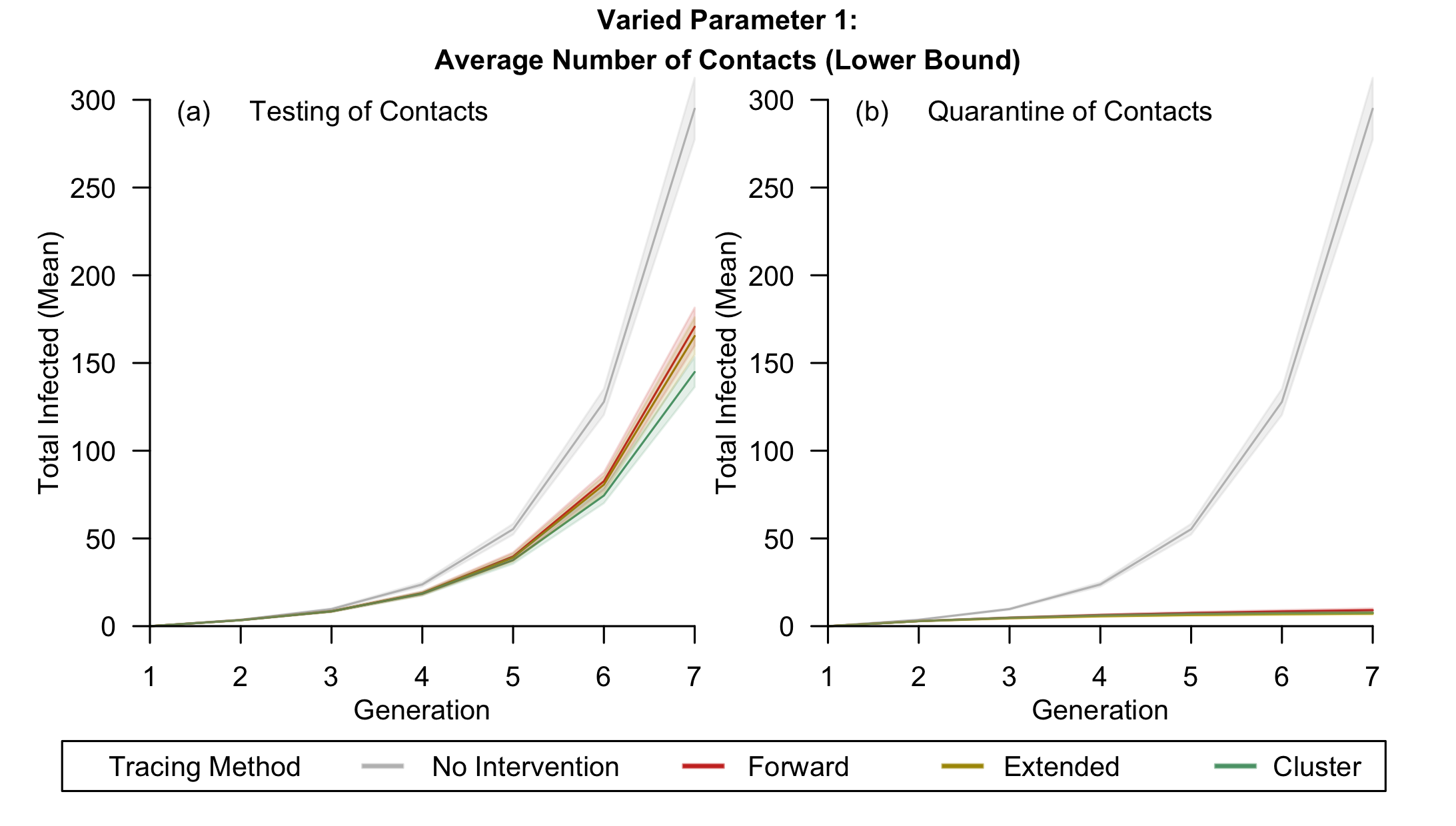


Supplementary Figure 7: Transmission across Generations under Varied Parameter 1.


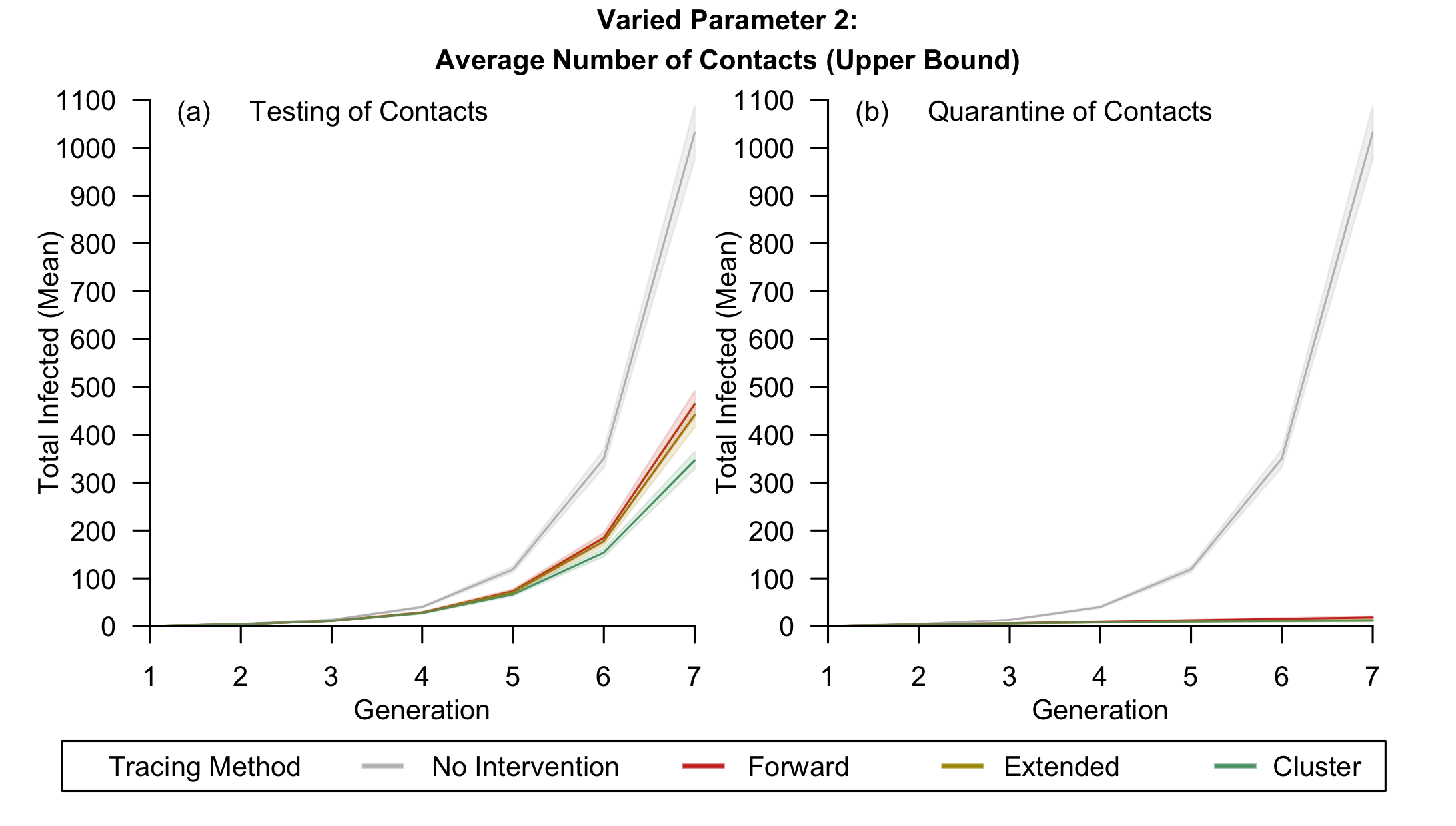


Supplementary Figure 8: Transmission across Generations under Varied Parameter 2.


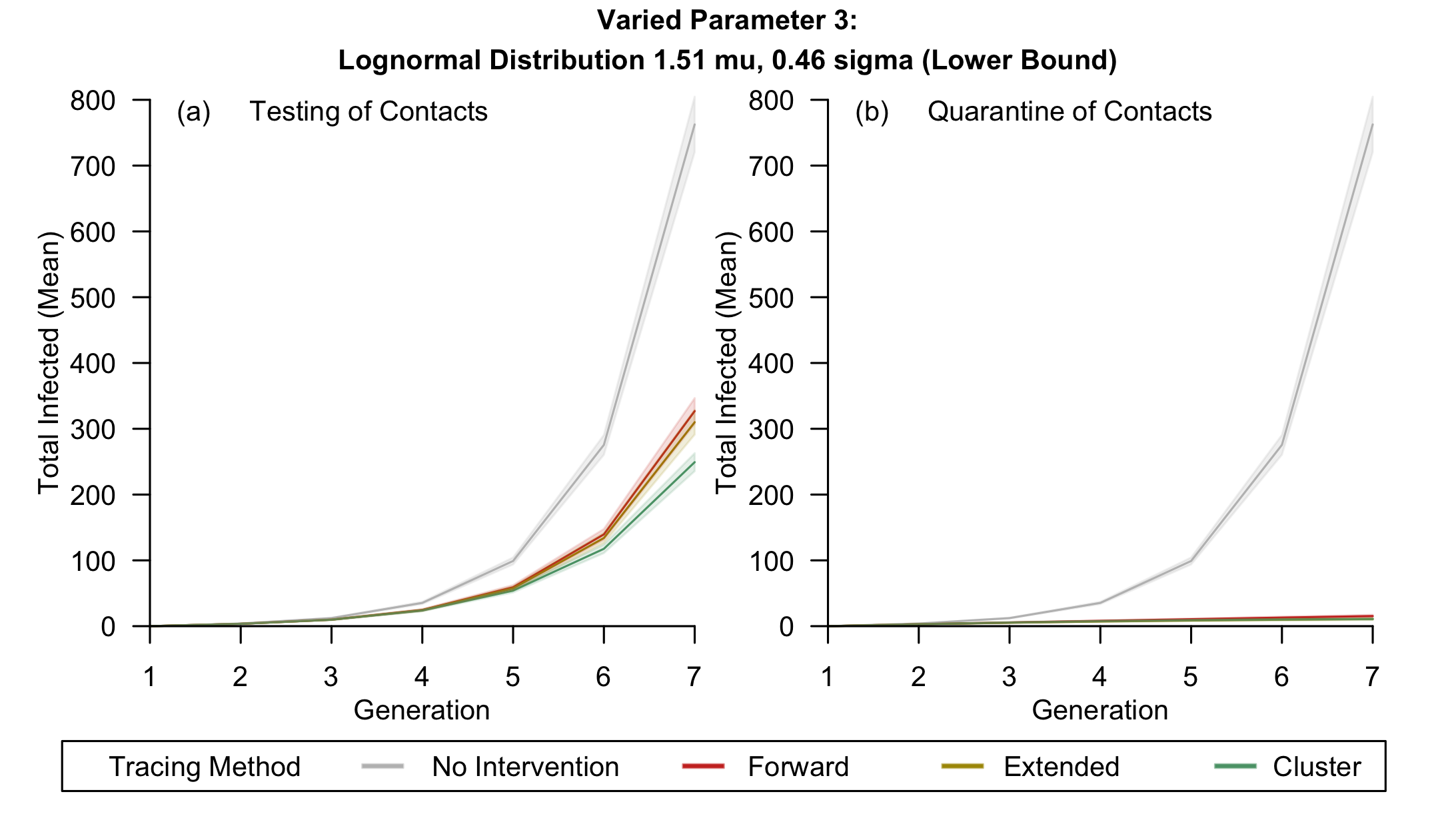


Supplementary Figure 9: Transmission across Generations under Varied Parameter 3.


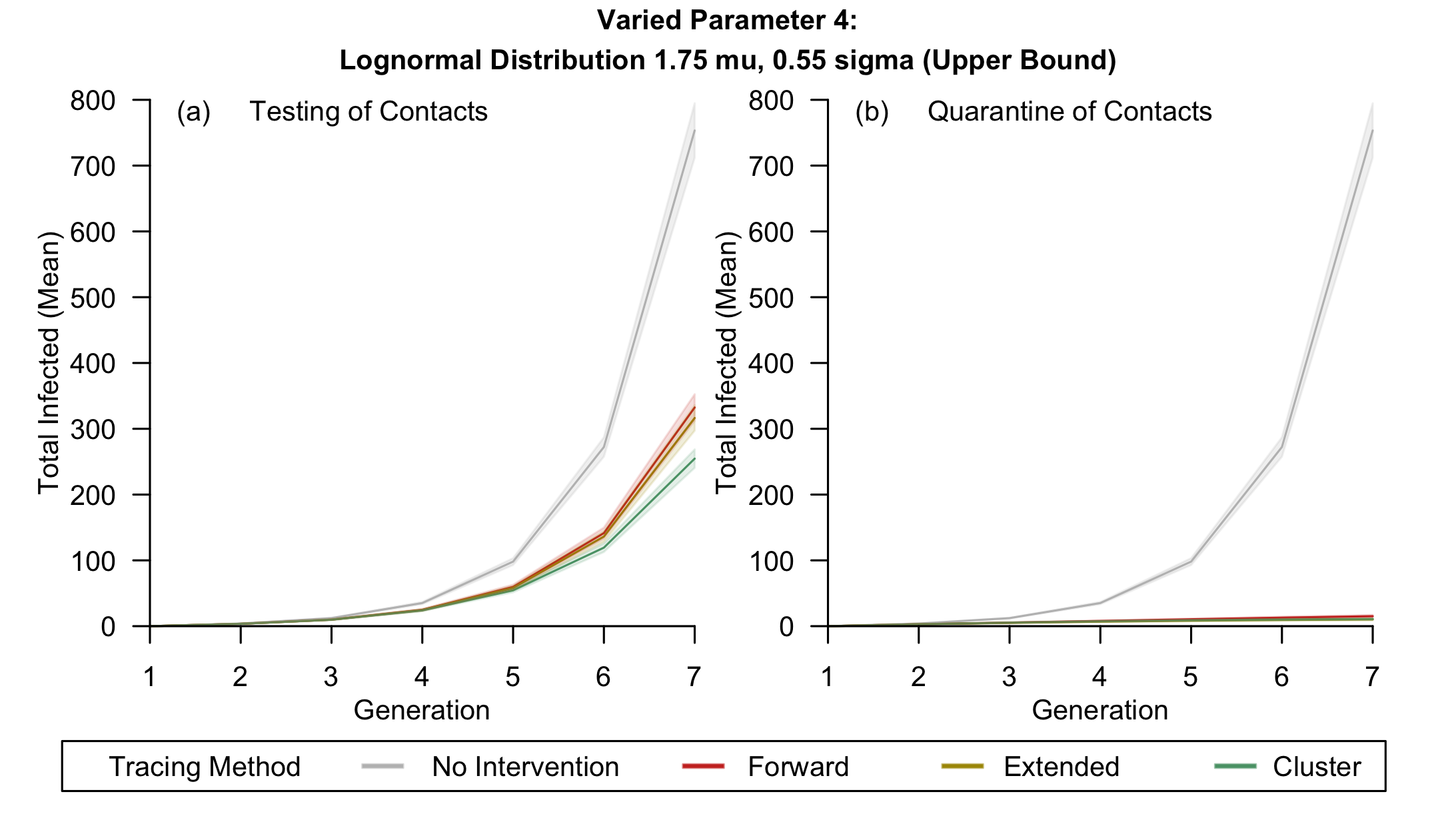


Supplementary Figure 10: Transmission across Generations under Varied Parameter 4.


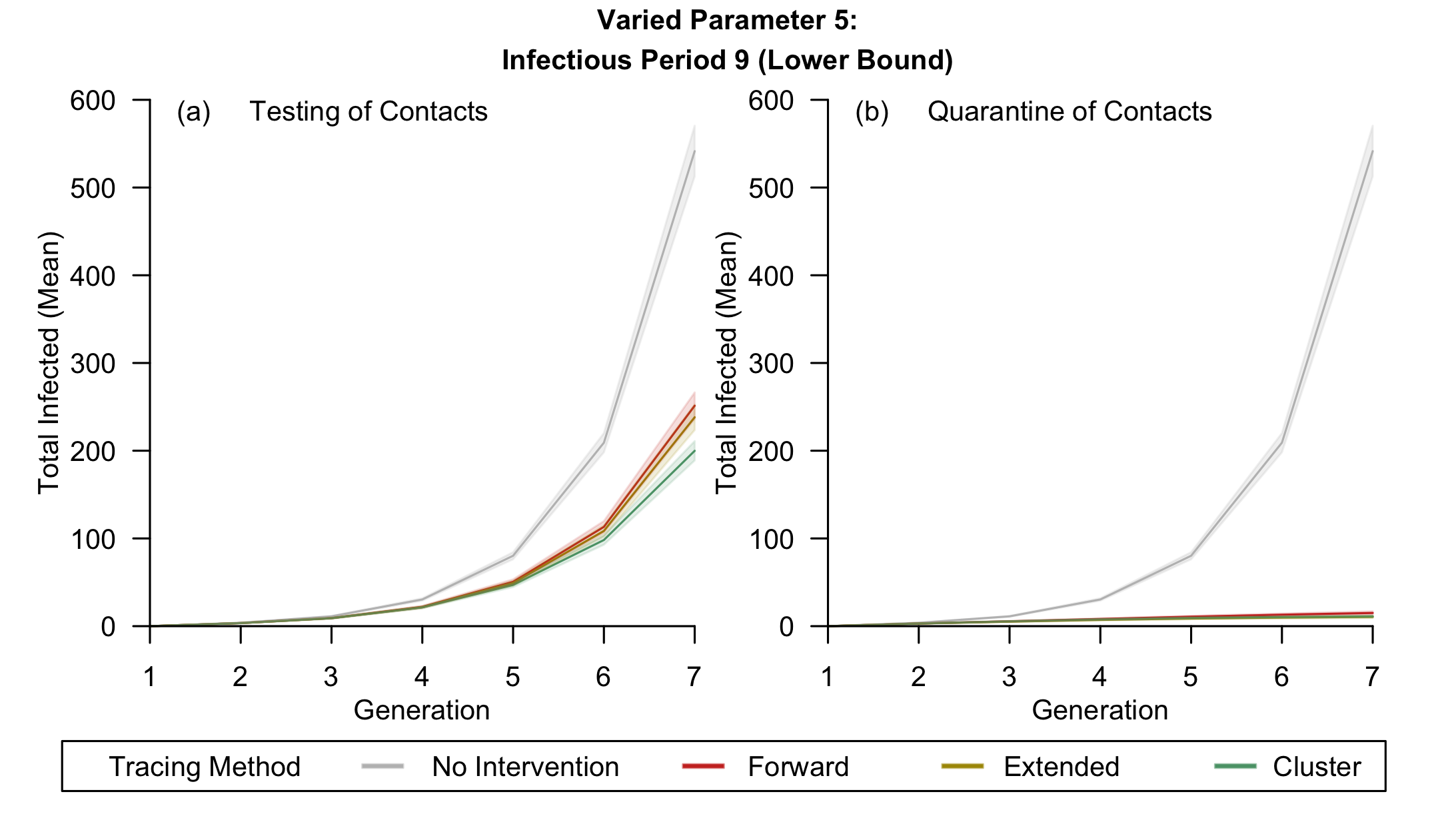


Supplementary Figure 11: Transmission across Generations under Varied Parameter 5.


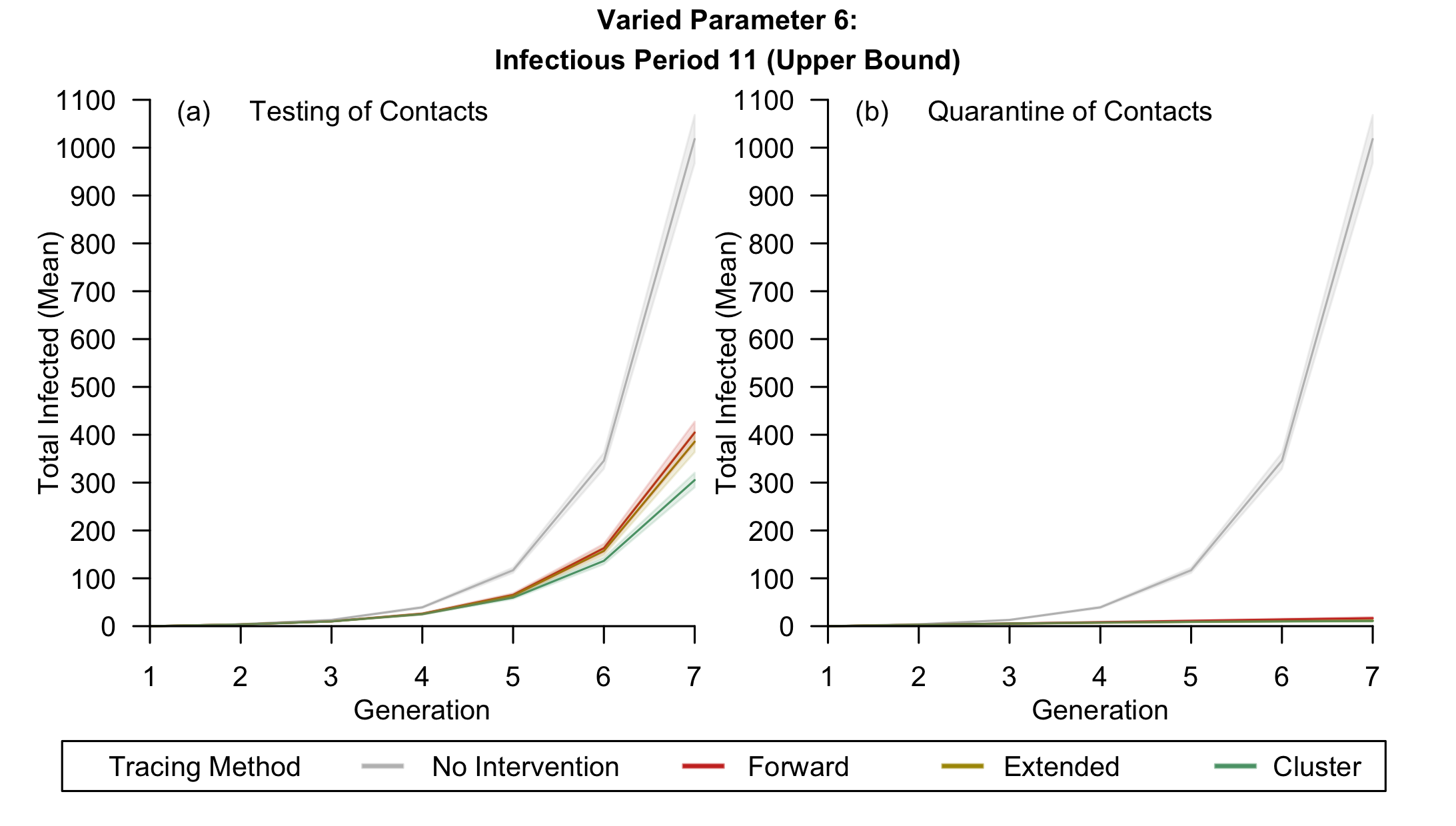


Supplementary Figure 12: Transmission across Generations under Varied Parameter 6.


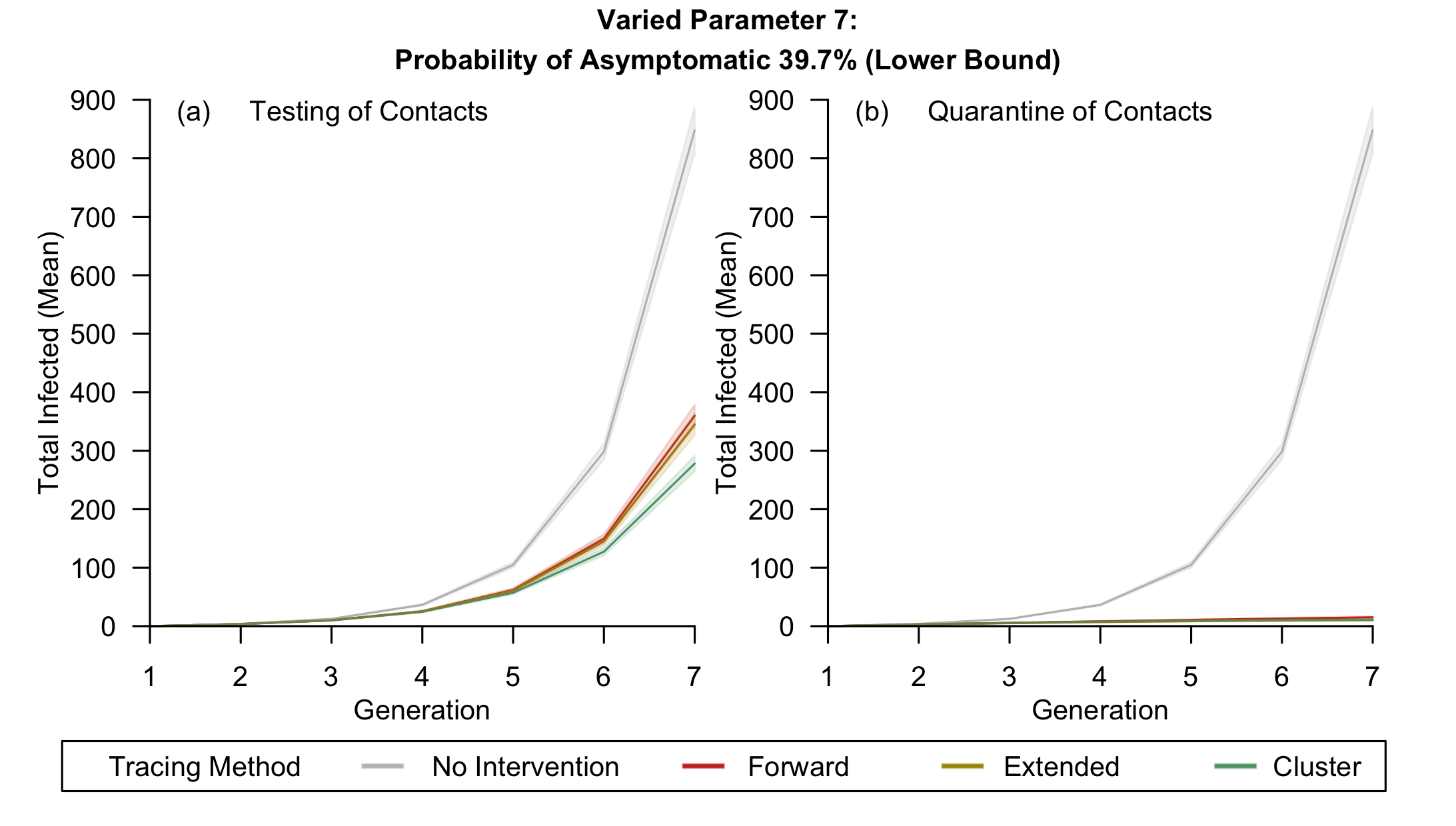


Supplementary Figure 13: Transmission across Generations under Varied Parameter 7.


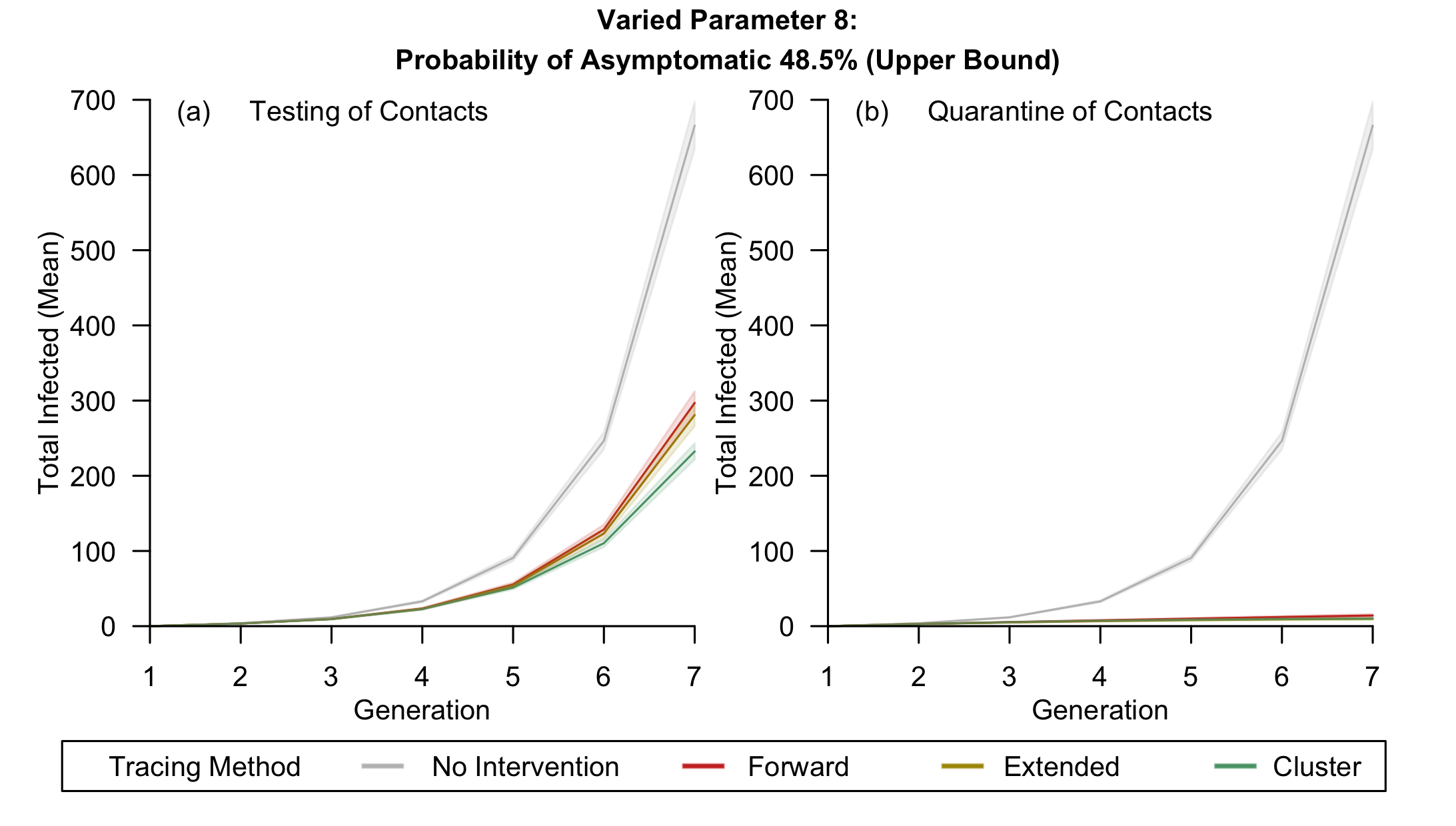


Supplementary Figure 14: Transmission across Generations under Varied Parameter 8.


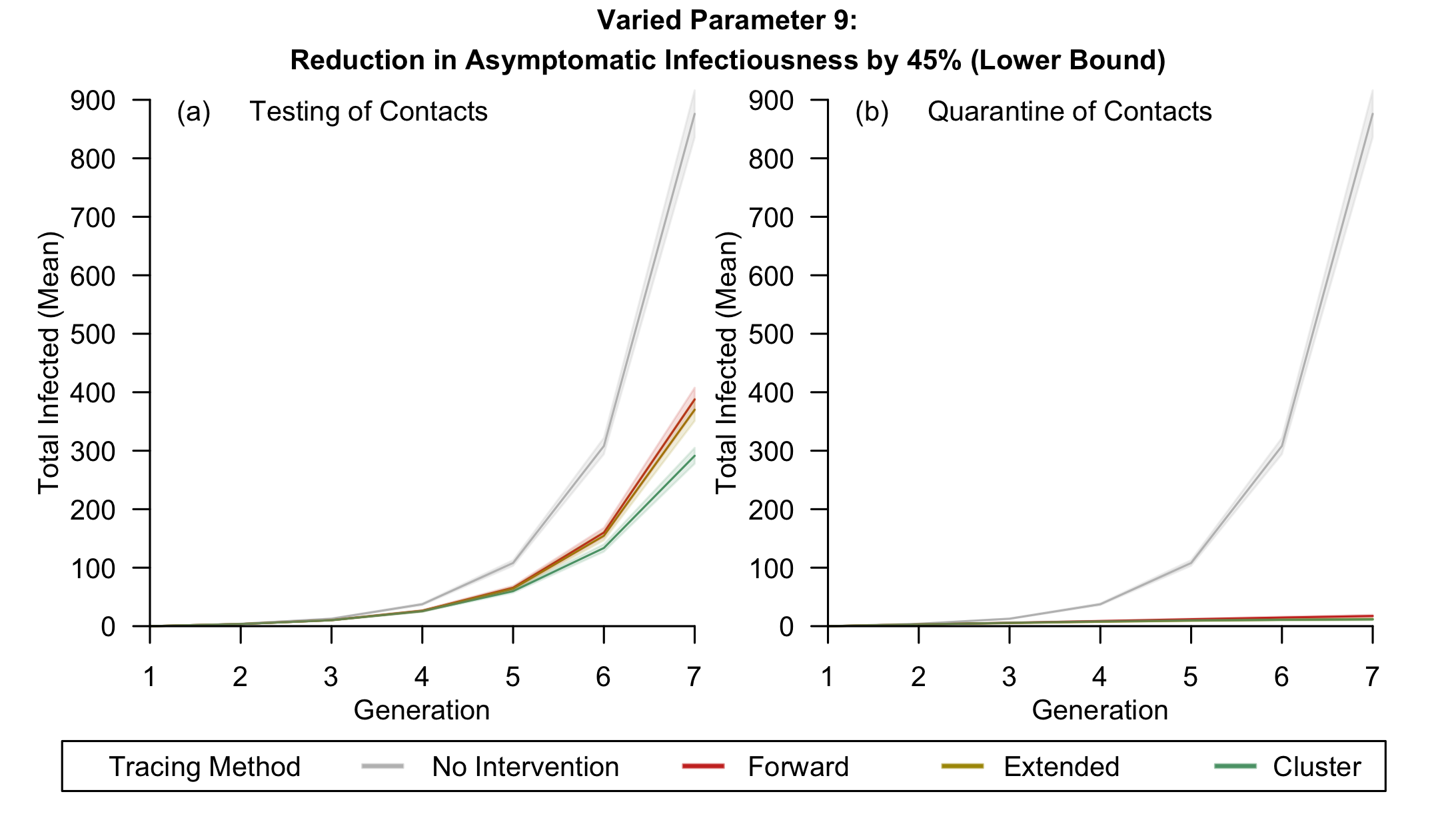


Supplementary Figure 15: Transmission across Generations under Varied Parameter 9.


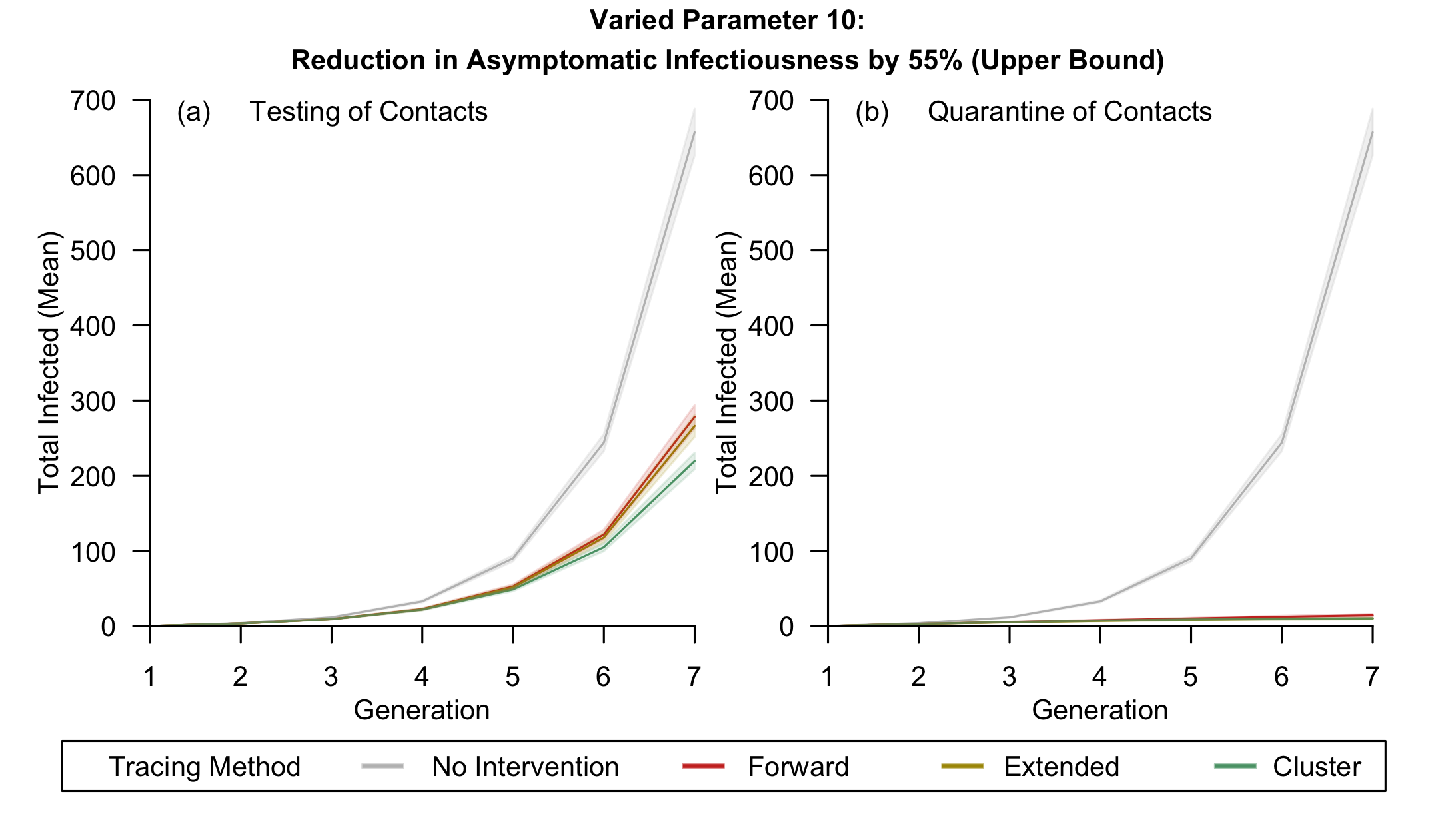


Supplementary Figure 16: Transmission across Generations under Varied Parameter 10.


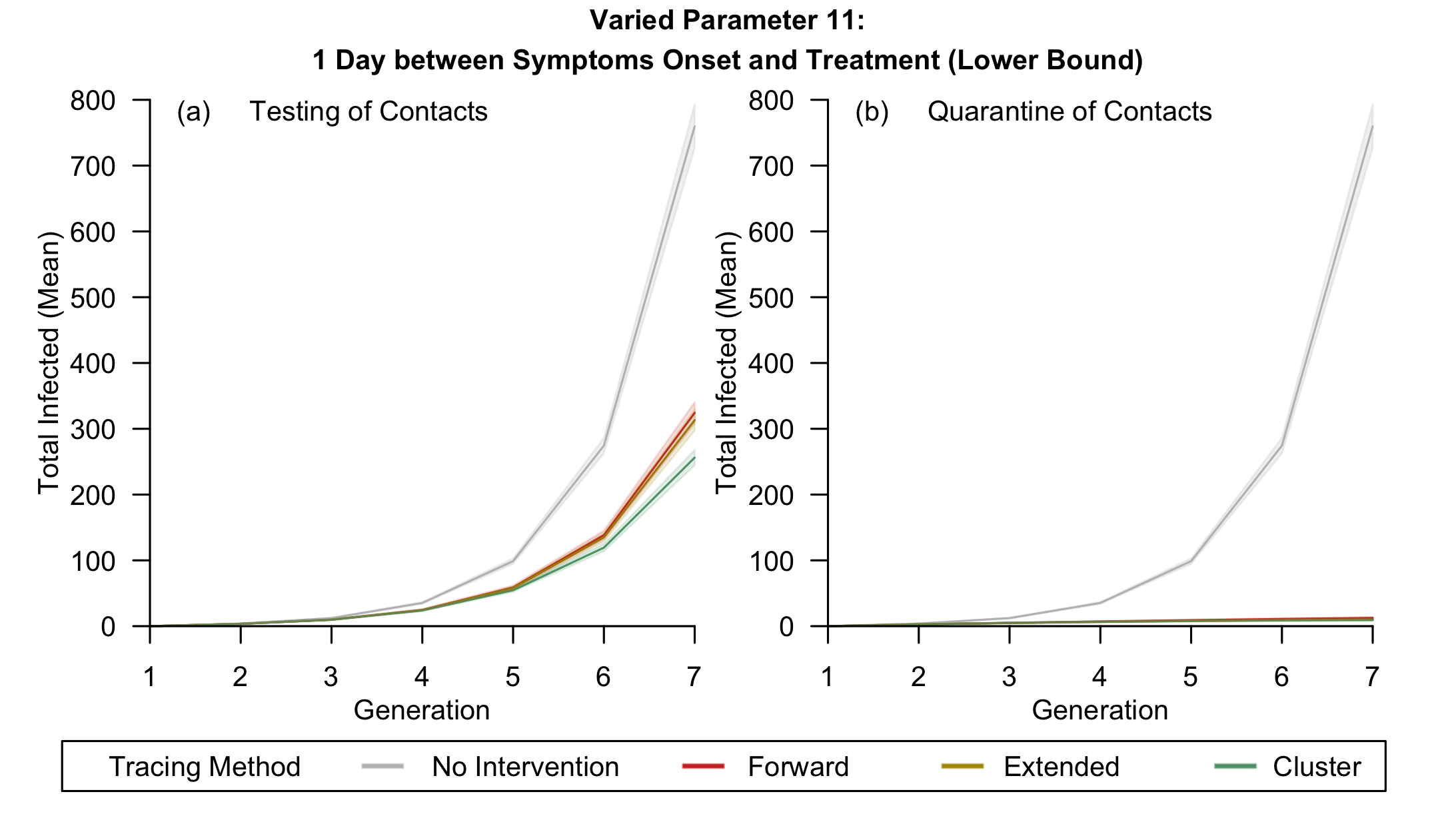


Supplementary Figure 17: Transmission across Generations under Varied Parameter 11.


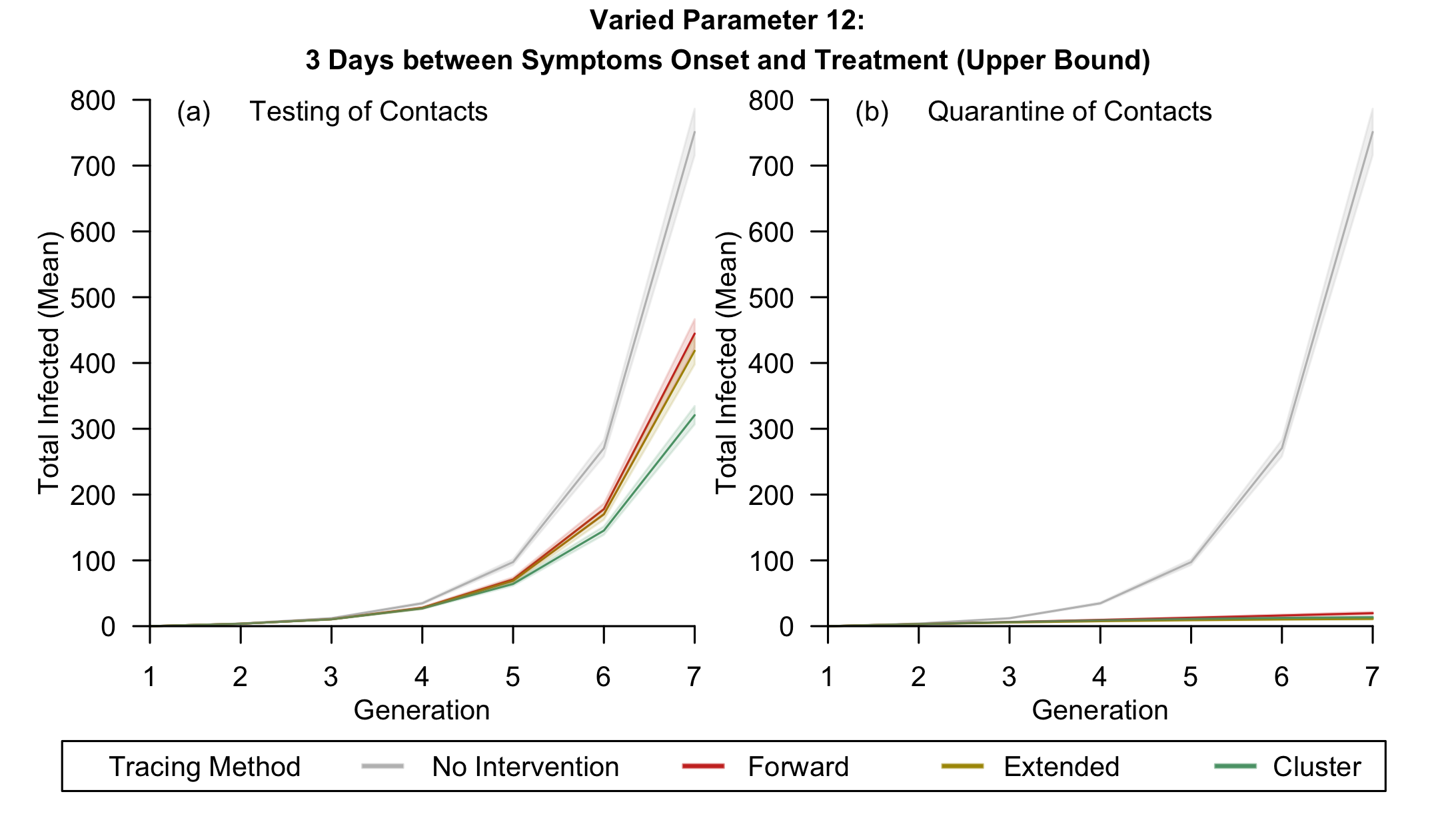


Supplementary Figure 18: Transmission across Generations under Varied Parameter 12.

## References

Buitrago-Garcia, D., Ipekci, A. M., Heron, L., Imeri, H., Araujo-Chaveron, L., Arevalo-Rodriguez, I., Ciapponi, A., Cevik, M., Hauser, A., Alam, M. I., Meili, K., Meyerowitz, E. A., Prajapati, N., Qiu, X., Richterman, A., Robles-Rodriguez, W. G., Thapa, S., Zhelyazkov, I., Salanti, G., & Low, N. (2022). Occurrence and transmission potential of asymptomatic and presymptomatic SARS-CoV-2 infections: Update of a living systematic review and meta-analysis. *PLOS Medicine*, *19*(5), e1003987. https://doi.org/10.1371/journal.pmed.1003987

Dhungel, B., Rahman, M. S., Rahman, M. M., Bhandari, A. K. C., Le, P. M., Biva, N. A., & Gilmour, S. (2022). Reliability of Early Estimates of the Basic Reproduction Number of COVID-19: A Systematic Review and Meta-Analysis. *International Journal of Environmental Research and Public Health*, *19*(18), 11613. https://doi.org/10.3390/ijerph191811613

McAloon, C., Collins, Á., Hunt, K., Barber, A., Byrne, A. W., Butler, F., Casey, M., Griffin, J., Lane, E., McEvoy, D., Wall, P., Green, M., O’Grady, L., & More, S. J. (2020). Incubation period of COVID-19: A rapid systematic review and meta-analysis of observational research. *BMJ Open*, *10*(8), e039652. https://doi.org/10.1136/bmjopen-2020-039652

Puhach, O., Meyer, B., & Eckerle, I. (2023). SARS-CoV-2 viral load and shedding kinetics. *Nature Reviews Microbiology*, *21*(3), Article 3. https://doi.org/10.1038/s41579-022-00822-w

Wang, B., Andraweera, P., Elliott, S., Mohammed, H., Lassi, Z., Twigger, A., Borgas, C., Gunasekera, S., Ladhani, S., & Marshall, H. S. (2023). Asymptomatic SARS-CoV-2 Infection by Age: A Global Systematic Review and Meta-analysis. *The Pediatric Infectious Disease Journal*, *42*(3), 232–239. https://doi.org/10.1097/INF.0000000000003791
